# Epidemiological and molecular analysis of anthrax cases of the Zhambyl region Kazakhstan in 2023

**DOI:** 10.3389/fpubh.2025.1620930

**Published:** 2025-07-28

**Authors:** Uinkul Izbanova, Altyn Rysbekova, Zauresh Zhumadilova, Galina Kovaleva, Gulnara Tokmurziyeva, Bek Abdeliyev, Duman Yessimseit, Elmira Begimbayeva, Saule Umarova, Anar Zarkymanova, Meruert Sabitova, Aisazhan Yussupov, Alexandr Shevtsov, Svetlana Isaeva, Nur Tukhanova

**Affiliations:** ^1^M. Aikimbayev’s National Scientific Center for Especially Dangerous Infections, National Holding “QazBioPharm”, Almaty, Kazakhstan; ^2^Department of Infectious and Tropical Disease, S. Asfendiyarov Kazakh National Medical University, Almaty, Kazakhstan

**Keywords:** anthrax, monitoring, epizootic, strain, Kazakhstan

## Abstract

**Background:**

Anthrax, caused by *Bacillus anthracis*, continues to pose a serious zoonotic threat in endemic areas such as southern Kazakhstan. Its persistence in the environment through resilient spores facilitates prolonged transmission cycles between animals and humans.

**Objective:**

This study aimed to characterize the epidemiological, clinical, and molecular features of human anthrax cases reported in the Zhambyl region of Kazakhstan during 2023.

**Methods:**

A total of 41 suspected cases were investigated, of which 19 were confirmed by culture and PCR (targeting pXO1 and pXO2). Data collection included patient demographics, exposure circumstances, clinical manifestations, and laboratory diagnostics. MLVA-31 genotyping was used to characterize *B. anthracis* isolates from culture-positive patients.

**Results:**

Confirmed cases were clustered in five district localities, yielding an incidence rate of 1.55 per 100,000 population. The majority of patients were male (84.2%), with exposure primarily linked to slaughter activities (68.4%). Culture was successful in 12 of 19 confirmed cases, and all isolates were susceptible to a broad range of antibiotics, including ciprofloxacin and doxycycline. Genotyping revealed three distinct clusters: one matching the A.Br.001/002 genotype common to northeastern China and Mongolia, and two divergent clusters likely represent localized evolution of *B. anthracis* strains. Five previously undocumented foci of anthrax were identified, suggesting wider regional spread than previously recognized.

**Conclusion:**

The 2023 outbreak of anthrax in the Zhambyl region highlights the ongoing risk posed by *B. anthracis*, particularly in areas with active livestock trade and conducive environmental conditions. The genetic diversity among isolates suggests both recent transmission and deeper endemic roots. Strengthening livestock vaccination programs, improving rural surveillance, and promoting awareness among high-risk populations are critical to preventing future outbreaks.

## Introduction

Anthrax is a disease caused by the spore-forming bacterium *Bacillus anthracis*. The key characteristic of *B. anthracis* is its ability to form spores, which enables the pathogen to persist in the environment for decades (1). Anthrax spores in soil are highly resistant and can cause disease when ingested by herbivorous animals. Once inside a living organism, the bacteria release potent toxins responsible for severe pathological effects in both humans and animals (2, 3). The most common clinical form in humans is the cutaneous form, which occurs through contact with infected animals or animal products containing spores (4, 5).

Anthrax continues to be reported in many regions worldwide, with varying incidence rates and seasonal patterns depending on environmental conditions, human-animal interactions, and the effectiveness of preventive measures (5, 6). However, anthrax outbreaks among humans continue to be recorded in endemic countries.

In 2023, the highest number of human anthrax cases were reported in Zambia (684 cases), Zimbabwe (412 cases), China (385 cases) and Indonesia (93 cases). Anthrax cases in neighboring countries to Kazakhstan was registered in Russia (19 cases), Kyrgyzstan (20 cases) and Tajikistan (80 cases) in 2023 and the main source of infection was represented by infected cattle (7).

Anthrax cases are reported every year in Kazakhstan especially in southern regions of Kazakhstan posing a significant challenge to both agricultural economies and public health.

On the territory of Kazakhstan in 2023, total 37 cases of human anthrax were registered (Zhambyl, Akmola and Karaganda regions), of which 1 case was fatal, the incidence rate was 0.18 per 100 thousand population (8). Among this, 19 cases of cutaneous anthrax were registered in Zhambyl region. Zhambyl region located in the southern part of Kazakhstan is adjacent to Ulytau, Turkestan, Almaty regions and south part with Kyrgyzstan. Annual incidence rates of anthrax from 2018 to 2023 ranged from 0.09 to 1.56 per 100,000 populations. The highest incidence was observed in 2023 (1.55; 95% CI: 0.90–2.30), followed by 2022 (1.13; 95% CI: 0.50–1.74) (Table 1). This is due to intensive animal husbandry and the presence of a combination of soil and climatic conditions favorable for the persistence of *B. anthracis* spores in the environment. The density of anthrax foci in Zhambyl region is significantly higher than in the northern and central regions of Kazakhstan (9, 10).

**Table 1:** Annual incidence of human anthrax cases in the Zhambyl region, Kazakhstan (2018–2023).

Year	Cases	Incidence rate (per 100,000)	95% CI
2018	1	0.09	0.00–0.27
2019	4	0.36	0.09–0.71
2020	1	0.09	0.00–0.27
2021	1	0.09	0.00–0.26
2022	13	1.13	0.50–1.74
2023	19	1.55	0.90–2.30

Number of reported cases, incidence rates per 100,000 population, and corresponding 95% confidence intervals.

This study aimed to determine the epidemiological, clinical, and molecular characteristics of human anthrax cases in Zhambyl region, Kazakhstan in 2023.

## Materials and methods

### Epidemiological data

Anthrax cases in humans in the Zhambyl region in 2023 were identified through the Zhambyl epidemiological surveillance system. Suspected anthrax reported cases further investigated a group of clinical, epidemiological and laboratory personnel with visit of the outbreak location to conduct investigations and implement disease control interventions. The epidemiologists conducted case-finding for suspected anthrax cases in affected areas, epidemiologically unfavorable area and analyzed their exposure history. The study included cases presenting with clinical signs and/or symptoms of cutaneous anthrax in combination with history of exposure and laboratory testing. All human anthrax cases were diagnosed according to the standardized case definitions established by the Clinical protocol for diagnosis and treatment of anthrax Kazakhstan Ministry of Health in 2016. A suspected case of the cutaneous anthrax is diagnosed in the presence of an acute illness characterized by high fever, a painless primary skin lesion with perifocal or widespread edema, at one of the following stages of development: papule, pustule (hemorrhagic), ulcer (flat, dry, with a black, dense eschar at the base, on an infiltrated foundation, surrounded by a hyperemic blister rim), black, dense eschar. A probable case of anthrax is diagnosed when the criteria for a suspected case are met, with at least one of the following: the patient has resided in or visited an anthrax-endemic area (where cases of anthrax in humans or animals have been reported) within 2 weeks prior to illness onset and has at least one of the following risk factors:

- Contact with animals or participation in the butchering of an infected animal.
- Preparation and consumption of inadequately cooked meat.
- Involvement in the procurement, transportation, or processing of animal products.
- Participation in the cleaning of facilities or areas where livestock are or were kept.
- Bites from blood-sucking insects.
- Participation in excavation or other soil-related activities.
- Contact with meat or animal hides brought from an anthrax-endemic area.
- Epidemiological link to a confirmed anthrax case.

A confirmed case of anthrax is diagnosed when an appropriate clinical specimen (such as pustular exudate, tissue sample from beneath the eschar) is tested and at least one of the following results:

- Culture of *B. anthracis* and;
- Positive PCR test and/or;
- Positive serological test (ELISA).

In all suspected anthrax cases, routine diagnostic testing including PCR and bacterial culture was performed at local public health laboratories in accordance with national protocols. Culture-positive samples were subsequently transported under appropriate biosafety conditions to the Aikimbayev’s National Scientific Center for Especially Dangerous Infections (NSCEDI) for confirmatory culture testing and advanced molecular genotyping. This additional analysis was conducted to characterize the *B. anthracis* isolates and support epidemiological investigations.

### Culture test and antibiotic susceptibility

Samples collected for laboratory testing included serum and cutaneous lesion samples, such as blister fluid obtained from skin lesions characteristic of cutaneous anthrax.

For culture method was used sheep blood agar 5%. The phenotypic properties of *B. anthracis* strains were studied according to methodological guidelines “Laboratory diagnostics of anthrax in humans and animals” (based on order of the Ministry of Health of the RK 01.10.2004 and Order of the Ministry of Agriculture of the RK 07.10.2004 No. 725/575 “On strengthening measures to prevent anthrax in the Republic of Kazakhstan”).

Antibiotic susceptibility testing was conducted using the disk diffusion method. Zone diameters were recorded in millimeters and categorized as Susceptible (S), Intermediate (I), or Resistant (R) based on Guideline Determination of Antimicrobial Susceptibility of Causative Agents (11).

### DNA extraction and PCR

DNA was isolated from culture positive samples using the QIAamp DNA Mini Kit according (QIAGEN) manual. The concentration and purity of the extracted DNA were assessed spectrophotometrically using a NanoDrop One instrument (Thermo Fisher Scientific, USA). Samples with A260/A280 ratios in the range of 1.8–2.0 were selected for further analysis. The samples were stored at −20°C.

PCR amplification was carried out in a reaction volume of 25 μL, containing 1 × buffer, 1.5–2.5 mM MgCl₂ (optimized for each locus), 200 μM of each dNTP, 0.25 μM of a fluorescently labeled forward primer and a conventional reverse primer, 1.25 U of Taq DNA polymerase (Thermo Scientific), and 5 μL of template DNA. Amplification was performed using a QuantStudio 5 thermal cycler (Applied Biosystems, USA). Optimized amplification conditions included an initial denaturation at 94°C for 5 min (1 cycle), followed by 35 cycles of denaturation at 94°C for 30 s, primer annealing at 55–65°C for 35 s, and extension at 72°C for 35 s, with a final extension at 72°C for 7 min. Amplification products were verified by electrophoresis on a 1.2% agarose gel followed by fluorescent detection.

The bacterial culture and DNA extraction were performed in a Biosafety level 3 (BSL-3) laboratory.

### MLVA-31 genotyping

Phylogenetic analysis of *B. anthracis* isolates was performed using Multiple-Locus Variable-Number Tandem Repeat Analysis-31 (MLVA-31), which is based on the amplification of eight VNTR loci: seven classical loci (vrrA, vrrB1, vrrB2, vrrC1, vrrC2, CG3, pXO1, and pXO2) and 24 additional loci, including BAMS and Bavntr markers (Supplementary Table 1) (12, 13). Primers for amplification were selected according to published protocols (14–16) and were synthesized with fluorescent labels (FAM, HEX, NED, ROX, TAMRA, VIC, Cy3). For each isolate, a numerical matrix was generated from the allelic profiles, reflecting the number of repeats at each locus. Only the numeric repeat values were used in subsequent analyses.

Based on the resulting distance matrix, a phylogenetic tree was constructed using the Unweighted Pair Group Method with Arithmetic Mean (UPGMA) method, implemented in the (Phylogenetic Analysis Using Parsimony, version 4.0) PAUP v4.0 software (17). The UPGMA method is widely used for clustering isolates with presumed clonal origin, as it is based on the average distance between all pairs of elements from different clusters.

The constructed tree was exported in Nexus format and visualized using FigTree v1.4.3 (18). The resulting dendrogram served as the basis for cluster analysis, enabling the identification of genetic relationships among the isolates and the delineation of stable genotypic groupings.

### Statistical analysis

Statistical analyses were performed using STATA version 17 (StataCorp, College Station, TX, USA). Descriptive statistics were used to summarize demographic and clinical data. Bivariate associations between risk factors and confirmed cases were assessed using Fisher’s exact test, as appropriate. Stratified analyses by gender, age group, and exposure route were performed to identify potential differences in risk. Incidence rates were calculated with 95% confidence intervals using Poisson distribution.

### Ethics statement

All procedures in this study adhered to the ethical standards of the local ethical committee. Samples (blood, cutaneous lesion samples) were collected from patients after obtaining their informed consent. The attending physician at the local hospital documented these consents. Positive samples patients’ data were anonymized. The informed consent and the study were approved by the local ethics committee of NSCEDI (protocol 1, 03.02.2023).

## Results

### Epidemiological data and case description

In 2023, 41 suspected cases of anthrax were registered in the Zhambyl region, 19 cases of cutaneous anthrax were confirmed by culture and PCR methods. Confirmed cases were registered in four districts and one city; Zhuali district—9 cases, Talas district—6 cases, Sarysu district—1 cases, Baizak district—1 case and in the city of Taraz—2 cases were associated with 11 exposure events (Table 2). The incidence rate of cutaneous anthrax in the Zhambyl region in 2023 was 1.55 (95% CI: 0.92–2.37) cases per 100,000 population. A total of 19 cases of cutaneous anthrax were confirmed by laboratory testing. PCR (targeting pXO1 and pXO2) confirmed *Bacillus anthracis* in all 19 cases. Culture-based identification was successful in 12 of these cases. The 12 culture-positive isolates were further analyzed by molecular genotyping.

**Table 2:** Confirmed human anthrax cases in the Zhambyl region, Kazakhstan in 2023.

District	Village	Previously anthrax reported area	2023	
			Human cases	Source of infections
Zhuali	Tasbastau	No	3	Unknown-1, sheep-2
	Karykorgan	No	1	Cattle-1
	Dikhan	No	2	Horse and sheep-1, cattle-1
	Amansay	No	1	Sheep-1
	Koltogan	Yes	2	Horse-2
Sarysu	Zhanaryk	Yes	1	Sheep-1
Talas	Karatau	Yes	2	Sheep-1, cattle-1
	Kaskabulak	Yes	1	Sheep-1
	Tamdy	No	3	Cattle-3
Baizak	Kyzyl Zhuldyz	Yes	1	Sheep-1
Taraz city	–	Yes	2	Horse-1, sheep-1
Total			19	Cattle-6, sheep-8, mix (sheep and horse)-1, horse-3, unknown-1

Distribution of cases by district and village, presence of previous anthrax reports, and suspected sources of infection.

Of the 19 confirmed cutaneus anthrax cases, 84.2% were male and 15.8% female. The mean age was 42.4 years (range: 16–68), with most cases between 36 and 55 years old. Participation in slaughter activities was the most frequently reported exposure route (68.4%), while 26.3% reported handling or consuming processed meat. Sheeps (42.1%) and cattle (31.6%) were the most commonly identified sources of infection. Occupations of registered cases were mainly unemployed 68.4% people. Most patients (84.2%) presented through self-referral to medical facilities, while 15.8% were detected via active surveillance (Table 3).

**Table 3:** Epidemiological characteristics of human cutaneous anthrax cases reported in 2023 in Zhambyl region (*n* = 19).

Characteristics		Frequency		Percentage (%)	
Gender (*n* = 19)	Male	16		84.2	
	Female	3		15.8	
Age group (*n* = 19)	15–35	7		36.8	
	36–55	8		42.1	
	56–68	4		21.1	
	Mean age	42.4			
	Range	16–68			
Route of Transmission (*n* = 19)	Risk factor				
	Processed meat	Yes 5		26.3	
		No 14		73.7	
	Participated in the slaughter	Yes 13		68.4	
		No 6		31.6	
	Unknown	Yes 1		5.3	
		No 18		94.7	
Source of Infection (*n* = 19)	Cattle	6		31.6	
	Small livestock	8		42.0	
	Horse	3		15.8	
	Mixed (horse+small livestock)	1		5.3	
	Unknown	1		5.3	
Confirmation test (*n* = 19)	*B. anthracis* culture positive	12		63.2	
	Real-time PCR (pXO1-/pXO2+)	19		36.8	
Detection Method (*n* = 19)	Self-referral to the hospital	16		84.2	
	Active household surveillance	3		15.8	
		Min	Max	Mean	SD
Duration Between Exposure, Onset, and Hospitalization (Days)	Between Exposure and Onset (*n* = 19)	2	15	6,4	3,9
	Between onset and surveillance system notification/Hospitalization (*n* = 19)	1	12	4,8	2,9

Bivariate analysis using Fisher’s exact test revealed that individuals who participated in animal slaughter had 2.6 times higher odds of being confirmed anthrax cases compared to those who did not, although this result was not statistically significant (*p* = 0.21). No significant association was found between processed meat consumption and case confirmation (*p* = 1.00) (Table 4).

**Table 4:** Bivariate analysis of selected risk factors for confirmed cutaneous anthrax cases (*n* = 19) compared to non-confirmed suspected cases (*n* = 22) in Zhambyl region, 2023.

Risk factor	Confirmed (*n* = 19)	Not confirmed (*n* = 22)	Odds ratio	*p*-value
Participated in slaughter	13	10	2.60	0.21
Did not participate	6	12	—	—
Processed meat consumption	5	6	0.95	1.00
No processed meat	14	16	—	—

Odds ratios and *p*-values calculated using Fisher’s exact test.

**Figure 1:**
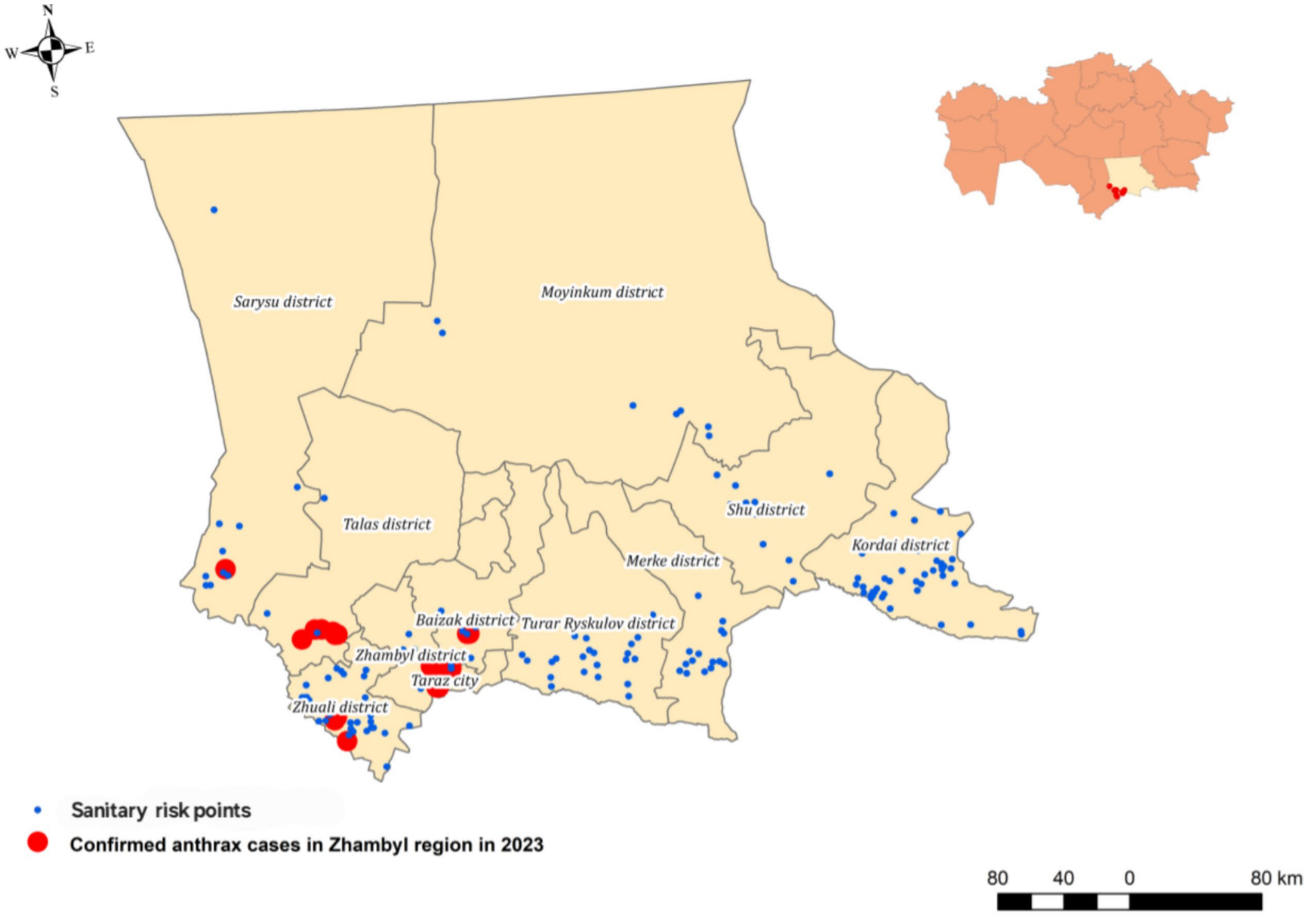
Map of the outbreak area showing sampling locations, and officially designated anthrax-risk areas (sanitary risk points).

The study identified five newly identified endemic foci of anthrax in the Zhambyl region in 2023, where the disease had not been previously reported (Figure 1). A retrospective analysis of the 2023 cutaneous anthrax cases in humans in the Zhambyl region was conducted to investigate the causes and contributing factors of the epidemiological situation. A chronological list of confirmed cutaneous anthrax cases was compiled (Table 5).

**Table 5:** A chronological list of confirmed human anthrax cases in Zhambyl region (*n* = 19) in 2023.

Patient/identification code	Gender	Age	Date of Illness	Living place	Occupation	Source of infection	Route of transmission	Confirmation test
Patient 1	M	40	18.05.23	Zhuali district, v. Koltogan	Teacher	Horse	Participated in the slaughter	PCR: positive Culture test: negative
Patient 2	M	55	24.05.23	Talas district, v. Tamdy	unemployed	Cattle	Participated in the slaughter	PCR: positive Culture test: positive
Patient 3	M	58	24.05.23	Talas district, v. Tamdy	unemployed	Cattle	Processed meat	PCR: positive Culture test: positive
Patient 4	M	50	30.05.23	Talas district, v. Tamdy	Security guard	Cattle	Participated in the slaughter	PCR: positive Culture test: positive
Patient 5	M	48	01.06.23	Zhuali district, v. Amansay	unemployed	Sheep	Processed meat	PCR: positive Culture test: negative
Patient 6	F	45	20.07.23	Zhuali district, v. Tasbastau	unemployed	Unknown	unknown	PCR: positive Culture test: negative
Patient 7	M	60	23.07.23	Baizak district, v. Kyzyl Zhuldyz	Meat seller	Sheep	Participated in the slaughter	PCR: positive Culture test: positive
Patient 8	M	68	28.07.23	Taraz city	Pensioner	Sheep	Participated in the slaughter	PCR: positive Culture test: positive
Patient 9	M	19	01.08.23	Zhuali district, v. Tasbastau	unemployed	Sheep	Participated in the slaughter	PCR: positive Culture test: negative
Patient 10	M	45	03.08.23	Zhuali district, v. Tasbastau	unemployed	Sheep	Participated in the slaughter	PCR: positive Culture test: positive
Patient 11	M	32	04.08.23	Zhuali district, v. Karykorgan	unemployed	Cattle	Participated in the slaughter	PCR: positive Culture test: negative
Patient 12	M	16	04.08.23	Zhuali district, v. Dikhan	student	Cattle	Participated in the slaughter	PCR: positive Culture test: negative
Patient 13	F	20	06.08.23	Talas district, Karatau	unemployed	Cattle	Processed meat	PCR: positive Culture test: positive
Patient 14	M	24	07.08.23	Taraz city	Meat seller	Horse	Participated in the slaughter	PCR: positive Culture test: positive
Patient 15	F	48	10.08.23	Talas district, Karatau	unemployed	Sheep	Processed meat	PCR: positive Culture test: negative
Patient 16	M	34	11.08.23	Talas district, v. Kaskabulak	unemployed	Sheep	Participated in the slaughter	PCR: positive Culture test: positive
Patient 17	M	36	13.09.23	Zhuali district, v. Dikhan	unemployed	Sheep and horse	Participated in the slaughter	PCR: positive Culture test: positive
Patient 18	M	34	30.09.23	Zhuali district, v. Koltogan	unemployed	Horse	Processed meat	PCR: positive Culture test: positive
Patient 19	M	46	09.10.23	Sarysu district, v. Zhanaryk	unemployed	Sheep	Participated in the slaughter	PCR: positive Culture test: positive

In 2023, human anthrax cases in the Zhambyl region exhibited seasonality, with multiple peaks observed in the end of May and August, the latter having the highest number of cases. From May to August, 63.2% of all reported anthrax cases occurred. Weather conditions played a significant role in shaping the epizootic and epidemiological patterns of anthrax. Human anthrax cases were recorded during the spring–summer period, with a peak in August, mirroring the seasonal patterns observed in livestock.

Laboratory testing confirmed *Bacillus anthracis* in clinical samples from 12 cases through culture method, while PCR (pXO1+/pXO2+) confirmed the diagnosis in all cases. None of the affected individuals had been vaccinated against anthrax. All 19 patients received antibiotic therapy with ciprofloxacin administered orally or IV for duration of 10–14 days, depending on clinical response according to national guidelines and antibiotic susceptibility. All patients were discharged with a fully recovery.

### Culture testing and antibiotic susceptibility

Among the 19 patients diagnosed with anthrax, bacterial cultures were positive in 12 cases. Antibiotic susceptibility testing was performed on all culture-positive samples. In the remaining 7 cases, cultures did not yield growth of *B. anthracis*. This is due to the fact that some patients had already begun antibiotic treatment before being admitted to the hospital, which can suppress bacterial growth and lead to negative culture results. These findings underline the importance of collecting clinical samples as early as possible, ideally before starting antimicrobial therapy.

The susceptibility of culture positive *B. anthracis* strains to antibiotics were assessed using the disk diffusion method. For determining *B. anthracis* susceptibility, antibacterial agents with specific concentrations recommended for the urgent prevention and treatment of anthrax were selected. The first-line antibiotics included benzylpenicillin, ampicillin, doxycycline, tetracycline, ciprofloxacin, and rifampicin.

The study results indicate that *B. anthracis* strains exhibit sensitivity to a broad spectrum of antibiotics, including benzylpenicillin, tetracycline, ampicillin, ciprofloxacin, rifampicin, kanamycin, erythromycin, gentamycin, streptomycin and doxycycline (Tables 5, 6).

**Table 6:** Results of susceptibility of *B. anthracis* to antibiotics.

Identification code/patient number	Antibiotics									
	Benzylpenicillin (10 μg)	Kanamycin (30 μg)	Rifampicin (5 μg)	Gentamicin (10 μg)	Erythromycin (15 μg)	Tetracycline (30 μg)	Ampicillin (10 μg)	Ciprofloxacin (5 μg)	Streptomycin (10 μg)	Doxycycline (30 μg)
	Susceptibility breakpoint (Zone Diameter, mm)									
	≥26	≥19	≥20	≥23	≥24	≥23	≥27	≥17	≥18	≥23
Zham-1-2023 Patient 2	37.2 S	27.8 S	27.1 S	29.1 S	25.8 S	36.3 S	35.8 S	37.8 S	23.8 S	35.5 S
Zham-11–2023 Patient 3	35.6 S	26.5 S	25.5 S	27.3 S	24.1 S	36.0 S	35.4 S	35.9 S	24.3 S	35.1 S
Zham-12-2023 Patient 4	37.9 S	27.2 S	22.8 S	24.4 S	25.0 S	37.1 S	34.9 S	35.0 S	21.9 S	36.8 S
Zham-13-2023 Patient 7	36.6 S	25.9 S	24.6 S	25.5 S	24.9 S	36.5 S	33.2 S	36.8 S	23.5 S	37.5 S
Zham-14-2023 Patient 8	36.6 S	30.6 S	26.1 S	29.1 S	23.8 S	36.3 S	35.8 S	37.6 S	24.0 S	37.5 S
Zham-15-2023 Patient 10	35.4 S	22.6 S	23.3 S	25.0 S	23.8 S	35.5 S	34.7 S	37.1 S	26.4 S	37.0 S
Zham-18-2023 Patient 14	35.3 S	27.3 S	27.0 S	24.4 S	32.3 S	36.6 S	37.3 S	35.4 S	22.9 S	36.1 S
Zham-19-2023 Patient 16	35 S	25.5 S	26.1 S	23.9 S	25.0 S	37.5 S	36.1 S	37.2 S	25.7 S	37.0 S
Zham-20-2023 Patient 13	33.3 S	26.4 S	25.9 S	25.5 S	24.8 S	36.0 S	37.5 S	36.9 S	24.1 S	35.8 S
Zham-22-2023 Patient 17	32.0 S	25.0 S	20.0 S	24.0 S	24.2 S	30.8 S	30.0 S	30.0 S	24.0 S	36.0 S
Zham-23-2023 Patient 18	32.0 S	25.0 S	20.0 S	25.0 S	24.2 S	30.8 S	30.0 S	30.0 S	25.0 S	36.0 S
Zham-24-2023 Patient 19	32.0 S	25.0 S	20.0 S	25.0 S	24.2 S	30.8 S	35.0 S	35.0 S	24.0 S	35.0 S

### MLVA-31 genotyping

Among the 12 positive culture samples, MLVA-31 genotyping was successfully performed 11 *Bacillus anthracis* isolates. The resulting profiles were compared with previously published regional genotypes from Kazakhstan. The Zhambyl isolates clustered into four distinct genotypes, grouped into two major clades. The first clade comprised eight isolates representing three genotypes, which differed at three VNTR loci. The second clade included a single genotype, which differed from the first clade by six VNTR loci.

Genotype 1 (Zham-15, Zham-18, Zham-20, Zham-23) included isolates obtained from patients 10, 13, 14, and 18, who resided in the villages of Tasbastau (Zhualy District), Karatau (Talas District), the city of Taraz, and the village of Koltogan (Zhuali District), respectively. Although these individuals had no known direct contact with each other, all were exposed to potentially contaminated animal products. Specifically, Patient 10 (Zham-15) participated in the slaughter of a sheep; Patient 14 (Zham-18) slaughtered a horse; Patient 13 (Zham-20) handled meat purchased from a local store; and Patient 18 (Zham-23) was involved in the slaughter of a horse. Despite the lack of direct epidemiological links and the geographic spread of the outbreak across approximately 70 to 168 km, the isolates were genetically identical according to MLVA-31 typing.

Genotype 2 (Zham-13, Zham-14) comprised isolates from Patients 7 and 8, who resided in the village of Kyzylzhuldyz (Baizak District) and the city of Taraz, respectively. Both patients were involved in the slaughter of clinically ill sheep as part of a single exposure event, which occurred in two locations approximately 6 km apart.

Genotype 3 (Zham-19, Zham-24) included isolates from Patients 16 and 19, residing in the villages of Kaskabulak (Talas District) and Zhanaryk (Sarysu District), respectively. Both individuals had slaughtered sheep prior to the onset of illness, though the exposures occurred at different times. Although these cases were not epidemiologically linked, the similarity in the nature of exposure despite differences in timing may suggest common environmental risk factors.

Genotype 4 (Zham-1, Zham-11, Zham-12) included isolates from patients 2, 3, and 4, all of whom were residents of the village of Tamdy (Talas District). These cases were directly linked to a single exposure event on May 20, involving the slaughter of a cow owned by patient 2. Patients 3 and 4 participated in this activity and subsequently developed symptoms (Supplementary Figure 1; Supplementary Tables 2, 3).

## Discussion

Anthrax cases are reported every year in Zhambyl region, in recent years the incidence of disease among humans and animals has been observed in the southern part of the region. The cluster of cutaneous anthrax cases in Zhambyl region during 2023, particularly in areas not previously marked as endemic, raises serious concerns about overlooked environmental reservoirs and gaps in local preventive infrastructure. Nineteen confirmed cases, scattered across four districts and the city of Taraz, point toward either a reactivation of dormant spores or new introductions via livestock movements. The event underlines the need for continuous field surveillance, particularly in seemingly low-risk areas.

All 19 cases were linked to participation in animal slaughter or handling of meat, aligning with what is typically known about anthrax exposure routes. The presence of skin lesions in patients, along with their documented exposure histories, served as the primary evidence for clinical diagnosis. It’s notable that over two-thirds of those infected were unemployed. This could reflect informal economic activities, such as backyard slaughter or unregulated meat processing areas that often escape oversight but carry high risk. The role of socio-economic status in exposure vulnerability may warrant closer examination in future field studies (10, 19).

The current epizootiological situation of anthrax in the Zhambyl region over the past 5 years has been characterized by a predominant incidence in cattle, accounting for 60.7% of cases. Despite sheep farming being the dominant form of agricultural production in these southern regions, cattle remain the primary species affected, suggesting potential gaps in livestock management and disease control strategies. The study revealed that the primary mode of transmission was direct contact with infected sheep or contaminated animal products, emphasizing the need for improved public awareness and preventive measures. Limited public health knowledge among the affected communities contributed to risky behaviors, such as butchering and consuming infected meat, further exacerbating the spread of the disease. Similar patterns have been observed in other anthrax-endemic regions, underscoring the necessity for targeted educational campaigns to mitigate human exposure risks (20, 21).

The seasonal occurrence of anthrax in livestock is influenced by the time of year. During winter, the ground remains covered with snow, limiting exposure to contaminated soil. In contrast, summer conditions increase the likelihood of animal contact with infected areas, leading to a rise in disease cases. During dry summer months, when dust levels are high, animals ingest large quantities of spores along with soil particles, further contributing to disease transmission. The Zhambyl region is characterized by dry, dusty pastures, where livestock almost exclusively become infected while grazing. Seasonality is most pronounced among sheep, primarily due to summer transhumance to distant pastures. The seasonal nature of the outbreak, with a peak in the summer months, is consistent with the well-documented seasonal patterns of anthrax in livestock, which are influenced by climatic conditions that facilitate spore persistence and transmission (20–22).

Vaccination of livestock remains a crucial measure for anthrax prevention. However, despite regular and widespread immunization efforts in high-risk epizootic areas, anthrax cases continue to be reported. Research findings indicate that uncontrolled migration of agricultural animals persists, along with incomplete livestock registration, leading to inadequate vaccination coverage against anthrax likely contributed to the re-emergence of anthrax in the Zhambyl region (23, 24). Despite routine vaccination programs in high-risk areas, the occurrence of anthrax cases indicates gaps in vaccination coverage and animal health monitoring. According to WHO recommendations, antibiotic prophylaxis may be a more effective strategy in non-endemic areas experiencing sudden outbreaks. This highlights the need for a more adaptive response strategy based on regional risk assessments.

The expansion of economic and trade relations between regions of Kazakhstan significantly increases the risk of introduction and spread of *B. anthracis* into neighboring areas. Genetic analysis supports the hypothesis that the outbreak originated from a persistent environmental reservoir. This situation was likely exacerbated by the movement of livestock between regions with a known history of anthrax cases. Unregulated animal migration, coupled with incomplete livestock registration and insufficient vaccination coverage, likely contributed to the registration of anthrax in the Zhambyl region (25).

All 19 cases were confirmed by PCR; however, cultures were successful in only 12 instances. This discrepancy between molecular and culture-based detection is due to factors such as prior antibiotic administration before hospital admission or a low concentration of viable bacteria in clinical specimens at the time of sampling. These findings highlight the limitations of culture in certain clinical scenarios and underscore the importance of using molecular diagnostics alongside traditional methods for accurate case confirmation. Still, the culture-positive isolates allowed for molecular work that added an important layer to the investigation.

MLVA-31 genotyping of *Bacillus anthracis* isolates from the Zhambyl region revealed four distinct genotypes, grouped into two closely related clusters. This pattern indicates the circulation of multiple, yet genetically related, strains within a relatively confined geographic area. Such a genetic structure is consistent with localized transmission dynamics and the presence of environmentally persistent spores (10). Notably, two of the four identified genotypes included strains associated with epidemiologically unrelated outbreaks. While homoplasy at VNTR loci cannot be excluded thereby limiting the ability to definitively attribute identical profiles to a single transmission chain the temporal proximity of these cases and complete identity across all 31 loci suggest the potential existence of unrecognized transmission pathways during the outbreak period in the Zhambyl region (26–28).

These findings highlight the importance of integrating high-resolution molecular genotyping with traditional field epidemiology to elucidate outbreak dynamics and trace sources of infection in endemic settings. The future application of whole-genome sequencing to isolates sharing identical MLVA-31 profiles but originating from different outbreaks may help resolve uncertainties related to VNTR homoplasy and substantially enhance the resolution and effectiveness of epidemiological investigations.

Limitations in the data should be addressed: the sample size is small, and while slaughter activities were linked to higher odds of infection, the association wasn’t statistically significant. Larger studies are needed to confirm behavioral risk factors and possibly trace environmental contamination.

This outbreak underscores that anthrax remains a public health concern in Central Asia. Better awareness, routine livestock vaccination, and improved disease reporting systems remain a critical component of anthrax prevention. The study findings indicate that many affected individuals were unaware of the risks associated with handling infected animals.

Furthermore, enhanced surveillance and early detection mechanisms should be prioritized to prevent future outbreaks. Implementing a more robust livestock tracking system, improving diagnostic capacities, can aid in mitigating the spread of anthrax in the region.

## Conclusion

The 2023 anthrax cases in the Zhambyl region highlights the ongoing challenges associated with anthrax control in endemic areas. Epidemiological findings emphasize the role of direct animal contact and seasonal influences, while molecular analysis confirms the genetic relatedness of the circulating *B. anthracis* strains to previously reported strains in the region. Addressing gaps in vaccination coverage, improving public health education, and strengthening disease surveillance will be critical in reducing the burden of anthrax in the future. A multifaceted approach that integrates veterinary, environmental, and public health interventions is necessary to mitigate the risk of future outbreaks.

## Data Availability

The original contributions presented in the study are included in the article/Supplementary material, further inquiries can be directed to the first author and/or corresponding author.
