# Supplementary material for: Epidemiological and molecular analysis of anthrax cases of the Zhambyl region Kazakhstan in 2023
Source: Front Public Health. 2025 Jul 28;13:1620930. doi: 10.3389/fpubh.2025.1620930 (PMC12336241; doi:10.3389/fpubh.2025.1620930)
Supplement: Supplementary file 1 [file Table_1.docx]

Supplementary table 1. Primers were used for amplification

| **Marker Locus** | **Forward Primer** | **Sequence (5'-3')** | **Fluorescent Label** | **Tm (°C)** |
| --- | --- | --- | --- | --- |
| vrrA | vrrA-f1-fam | CACAACTACCACCGATGGCACA | Fam | 71.0 |
|  | vrrA-r1 | GCGCGTTTCGTTTGATTCATAC | None | 69.7 |
| vrrB1 | vrrB1-f1-fam | ATAGGTGGTTTTCCGCAAGTTATTC | Fam | 70.0 |
|  | vrrB1-r1 | GATGAGTTTGATAAAGAATAGCCTGTG | None | 69.0 |
| vrrB2 | vrrB2-f1-fam | CACAGGCTATTCTTTATCAAACTCATC | Fam | 72.0 |
|  | vrrB2-r1 | CCCAAGGTGAAGATTGTTGTTGA | None | 68.8 |
| vrrC1 | vrrC1-f1 | GAAGCAAGAAAGTGATGTAGTGGAC | None | 66.8 |
|  | vrrC1-r1-fam | CATTTCCTCAAGTGCTACAGGTTC | Fam | 67.5 |
| vrrC2 | vrrC2-f1-hex | CCAGAAGAAGTGGAACCTGTAGCAC | Hex | 70.9 |
|  | vrrC2-r1 | GTCTTTCCATTAATCGCGCTCTATC | None | 70.6 |
| CG3 | CG3-f1-ned | TGTCGTTTTACTTCTCTCTCCAATAC | Ned | 66.2 |
|  | CG3-r1 | AGTCATTGTTCTGTATAAAGGGCAT | None | 66.2 |
| pXO1-aat | pXO1-AAT-f3-fam | CAATTTATTAACGATCAGATTAAGTTCA | Fam | 66.3 |
|  | pXO1-AAT-r3 | TCTAGAATTAGTTGCTTCATAATGGC | None | 66.7 |
| pXO2-at | pXO2-AT-f1-hex | TCATCCTCTTTTAAGTCTTGGGT | Hex | 64.4 |
|  | pXO2-AT-r1 | GTGTGATGAACTCCGACGACA | None | 65.8 |
| BAMS01 | BAMS01-f1-fam | GTTGAGCATGAGAGGTACCTTGTCCTTTTT | Fam | 70.2 |
|  | BAMS01-r1 | AGTTCAAGCGCCAGAAGGTTATGAGTTATC | None | 68.3 |
| BAMS03 | BAMS03-f1-vic | GCAGCAACAGAAAACTTCTCTCCAATAACA | Vic | 71.5 |
|  | BAMS03-r1 | TCCTCCCTGAGAACTGCTATCACCTTTAAC | None | 68.9 |
| BAMS05 | BAMS05-f1-rox | GCAGGAAGAACAAAAGAAACTAGAAGAGCA | Rox | 70.7 |
|  | BAMS05-r1 | ATTATTAGCAGGGGCCTCTCCTGCATTACC | None | 69.2 |
| BAMS13 | BAMS13-f1-vic | AATTGAGAAATTGCTGTACCAAACT | Vic | 66.8 |
|  | BAMS13-r1 | CTAGTGCATTTGACCCTAATCTTGT | None | 66.2 |
| BAMS15 | BAMS15-f1-fam | GTATTTCCCCCAGATACAGTAATCC | Fam | 67.9 |
|  | BAMS15-r1 | GTGTACATGTTGATTCATGCTGTTT | None | 67.4 |
| BAMS21 | BAMS21-f1-vic | TGTAGTGCCAGATTTGTCTTCTGTA | Vic | 69.1 |
|  | BAMS21-r1 | CAAATTTTGAGATGGGAGTTTTACT | None | 68.5 |
| BAMS22 | BAMS22-f1-fam | ATCAAAAATTCTTGGCAGACTGA | Fam | 66.4 |
|  | BAMS22-r1 | ACCGTTAATTCACGTTTAGCAGA | None | 65.7 |
| BAMS23 | BAMS23-f1-tamra | CGGTCTGTCTCTATTATTCAGTGGT | Tamra | 70.6 |
|  | BAMS23-r1 | CCTGTTGCTCCTAGTGATTTCTTAC | None | 69.3 |
| BAMS24 | BAMS24-f1-rox | CTTCTACTTCCGTACTTGAAATTGG | Rox | 69.9 |
|  | BAMS24-r1 | CGTCACGTACCATTTAATGTTGTTA | None | 69.1 |
| BAMS25 | BAMS25-f1-rox | CCGAATACGTAAGAAATAAATCCAC | Rox | 68.4 |
|  | BAMS25-r1 | TGAAAGATCTTGAAAAACAAGCATT | None | 67.9 |
| BAMS28 | BAMS28-f1-rox | CTCTGTTGTAACAAAATTTCCGTCT | Rox | 68.8 |
|  | BAMS28-r1 | TATTAAACCAGGCGTTACTTACAGC | None | 67.3 |
| BAMS30 | BAMS30-f1-vic | GCATAATCACCTACAACACCTGGTA | Vic | 68.9 |
|  | BAMS30-r1 | CAGAAAATATTGGACCTACCTTCC | None | 67.6 |
| BAMS31 | BAMS31-f1-tamra | GCTGTATTTATCGAGCTTCAAAATCT | Tamra | 69.1 |
|  | BAMS31-r1 | GGAGTACTGTTTGTTGAATGTTGTTT | None | 68.2 |
| BAMS34 | BAMS34-f1-tamra | CAGCAAAATCAATCGAATCAAA | Tamra | 67.3 |
|  | BAMS34-r1 | TGTGCTAAATCATCTTGCTTGG | None | 66.7 |
| BAMS44 | BAMS44-f1-vic | GCGAATTAATTGCTCCTCAAAT | Vic | 67.5 |
|  | BAMS44-r1 | GCACTTGAATATTTGGCGGTAT | None | 67.0 |
| BAMS51 | BAMS51-f1-rox | ATTTCCTGAAGCAGGTTGTGTT | Rox | 68.4 |
|  | BAMS51-r1 | TGCATCTAACAATGCAGAACAA | None | 67.3 |
| BAMS53 | BAMS53-f1-tamra | GAGGTGTGTTAGGTGGGCTTAC | Tamra | 68.9 |
|  | BAMS53-r1 | CATATTTTCACCTTAATTTTGGAAG | None | 68.0 |
| Bavntr12 | Bavntr12-f1-cy3 | CGTACGAAGTAGAAGTCATTAA | Cy3 | 66.5 |
|  | Bavntr12-r1 | GCATATAATTGCACCTCATCTAG | None | 66.1 |
| Bavntr16 | Bavntr16-f1-fam | CTCTTGAAAATATAAAACGCA | Fam | 65.9 |
|  | Bavntr16-r1 | GAATAATAAGGGTTCTCATGGTAT | None | 65.6 |
| Bavntr17 | Bavntr17-f1-cy3 | TAGGTAAACAAATTTTCGTAATC | Cy3 | 66.8 |
|  | Bavntr17-r1 | GATCGTACAACAGCAATTATCAT | None | 66.0 |
| Bavntr19 | Bavntr19-f1-fam | GTGATGAAATCGGACAAGTTAGGAG | Fam | 67.7 |
|  | Bavntr19-r1 | GAAATATTTTATTAAACATGCTTTCCATCC | None | 66.9 |
| Bavntr23 | Bavntr23-f1-rox | TTTAGAAACGTTATCACGCTTA | Rox | 66.6 |
|  | Bavntr23-r1 | GTAATACGTATGGTTCATTCCC | None | 65.9 |
| Bavntr35 | Bavntr35-f1-rox | AAATAATATGTTCCTTTTGCTG | Rox | 65.8 |
|  | Bavntr35-r1 | GTCCTGAAATAAATGCTGAAT | None | 65.3 |
