# Supplementary material for: Epidemiological and molecular analysis of anthrax cases of the Zhambyl region Kazakhstan in 2023
Source: Front Public Health. 2025 Jul 28;13:1620930. doi: 10.3389/fpubh.2025.1620930 (PMC12336241; doi:10.3389/fpubh.2025.1620930)
Supplement: Supplementary file 2 [file Table_2.docx]

Supplementary table 2. Amplicon sizes (in base pairs) for each VNTR locus, determined using GeneMapper software (Capillary electrophoresis, SeqStudio, Applied Biosystems).

| **Marker locus** | **Zham_1** | **Zham_11** | **Zham_12** | **Zham_13** | **Zham_14** | **Zham_15** | **Zham_18** | **Zham_19** | **Zham_20** | **Zham_23** | **Zham_24** |
| --- | --- | --- | --- | --- | --- | --- | --- | --- | --- | --- | --- |
| vrrA | 314 | 314 | 314 | 314 | 326 | 314 | 314 | 314 | 314 | 314 | 314 |
| vrrB1 | 238 | 238 | 238 | 229 | 229 | 238 | 238 | 238 | 238 | 238 | 238 |
| vrrB2 | 162 | 162 | 162 | 162 | 162 | 162 | 162 | 162 | 162 | 162 | 162 |
| vrrC1 | 571 | 571 | 571 | 607 | 607 | 607 | 607 | 607 | 607 | 607 | 607 |
| vrrC2 | 532 | 532 | 532 | 604 | 604 | 604 | 604 | 604 | 604 | 604 | 604 |
| CG3 | 158 | 158 | 158 | 153 | 153 | 153 | 153 | 153 | 153 | 153 | 153 |
| pXO1 | 129 | 129 | 129 | 132 | 132 | 132 | 132 | 132 | 132 | 132 | 132 |
| pXO2 | 139 | 139 | 139 | 135 | 135 | 135 | 135 | 137 | 135 | 135 | 137 |
| BAMS01 | 422 | 422 | 422 | 422 | 422 | 422 | 422 | 422 | 422 | 422 | 422 |
| BAMS03 | 624 | 624 | 624 | 624 | 624 | 624 | 624 | 624 | 624 | 624 | 624 |
| BAMS05 | 346 | 346 | 346 | 385 | 385 | 385 | 385 | 385 | 385 | 385 | 385 |
| BAMS13 | 384 | 384 | 384 | 474 | 474 | 474 | 474 | 474 | 474 | 474 | 474 |
| BAMS15 | 616 | 616 | 616 | 607 | 607 | 607 | 607 | 607 | 607 | 607 | 607 |
| BAMS21 | 676 | 676 | 676 | 676 | 676 | 676 | 676 | 676 | 676 | 676 | 676 |
| BAMS22 | 735 | 735 | 735 | 735 | 735 | 735 | 735 | 735 | 735 | 735 | 735 |
| BAMS23 | 651 | 651 | 651 | 651 | 651 | 651 | 651 | 651 | 651 | 651 | 651 |
| BAMS24 | 591 | 591 | 591 | 591 | 591 | 591 | 591 | 591 | 591 | 591 | 591 |
| BAMS25 | 391 | 391 | 391 | 391 | 391 | 391 | 391 | 391 | 391 | 391 | 391 |
| BAMS28 | 491 | 491 | 491 | 491 | 491 | 491 | 491 | 491 | 491 | 491 | 491 |
| BAMS30 | 828 | 828 | 828 | 864 | 864 | 864 | 864 | 864 | 864 | 864 | 927 |
| BAMS31 | 771 | 771 | 771 | 771 | 771 | 771 | 771 | 771 | 771 | 771 | 771 |
| BAMS34 | 423 | 423 | 423 | 423 | 423 | 423 | 423 | 423 | 423 | 423 | 423 |
| BAMS44 | 423 | 423 | 423 | 423 | 423 | 423 | 423 | 423 | 423 | 423 | 423 |
| BAMS51 | 493 | 493 | 493 | 493 | 493 | 493 | 493 | 493 | 493 | 493 | 493 |
| BAMS53 | 236 | 236 | 236 | 236 | 236 | 236 | 236 | 236 | 236 | 236 | 236 |
| Bavntr12 | 115 | 115 | 115 | 115 | 115 | 115 | 115 | 115 | 115 | 115 | 115 |
| Bavntr16 | 273 | 273 | 273 | 273 | 273 | 273 | 273 | 273 | 273 | 273 | 273 |
| Bavntr17 | 450 | 450 | 450 | 386 | 386 | 386 | 386 | 386 | 386 | 386 | 386 |
| Bavntr19 | 99 | 99 | 99 | 99 | 99 | 99 | 99 | 99 | 99 | 99 | 99 |
| Bavntr23 | 197 | 197 | 197 | 197 | 197 | 197 | 197 | 197 | 197 | 197 | 197 |
| Bavntr35 | 109 | 109 | 109 | 109 | 109 | 109 | 109 | 109 | 109 | 109 | 109 |
