# Supplementary material for: Epidemiological and molecular analysis of anthrax cases of the Zhambyl region Kazakhstan in 2023
Source: Front Public Health. 2025 Jul 28;13:1620930. doi: 10.3389/fpubh.2025.1620930 (PMC12336241; doi:10.3389/fpubh.2025.1620930)
Supplement: Supplementary file 3 [file Table_3.docx]

Supplementary table 3. Allelic profiles of 11 *Bacillus anthracis* isolates based on 31 VNTR loci (number of repeats)

| **Marker locus** | **Zham_1** | **Zham_11** | **Zham_12** | **Zham_13** | **Zham_14** | **Zham_15** | **Zham_18** | **Zham_19** | **Zham_20** | **Zham_23** | **Zham_24** |
| --- | --- | --- | --- | --- | --- | --- | --- | --- | --- | --- | --- |
| vrrA | 4 | 4 | 4 | 4 | 4 | 4 | 4 | 4 | 4 | 4 | 4 |
| vrrB1 | 9 | 9 | 9 | 8 | 8 | 9 | 9 | 9 | 9 | 9 | 9 |
| vrrB2 | 3 | 3 | 3 | 3 | 3 | 3 | 3 | 3 | 3 | 3 | 3 |
| vrrC1 | 35 | 35 | 35 | 45 | 45 | 45 | 45 | 45 | 45 | 45 | 45 |
| vrrC2 | 17 | 17 | 17 | 21 | 21 | 21 | 21 | 21 | 21 | 21 | 21 |
| CG3 | 2 | 2 | 2 | 1 | 1 | 1 | 1 | 1 | 1 | 1 | 1 |
| pXO1 | 8 | 8 | 8 | 8 | 8 | 8 | 8 | 8 | 8 | 8 | 8 |
| pXO2 | 8 | 8 | 8 | 6 | 6 | 6 | 6 | 7 | 6 | 6 | 7 |
| BAMS01 | 13 | 13 | 13 | 13 | 13 | 13 | 13 | 13 | 13 | 13 | 13 |
| BAMS03 | 30 | 30 | 30 | 30 | 30 | 30 | 30 | 30 | 30 | 30 | 30 |
| BAMS05 | 6 | 6 | 6 | 7 | 7 | 7 | 7 | 7 | 7 | 7 | 7 |
| BAMS13 | 20 | 20 | 20 | 30 | 30 | 30 | 30 | 30 | 30 | 30 | 30 |
| BAMS15 | 46 | 46 | 46 | 45 | 45 | 45 | 45 | 45 | 45 | 45 | 45 |
| BAMS21 | 10 | 10 | 10 | 10 | 10 | 10 | 10 | 10 | 10 | 10 | 10 |
| BAMS22 | 16 | 16 | 16 | 16 | 16 | 16 | 16 | 16 | 16 | 16 | 16 |
| BAMS23 | 11 | 11 | 11 | 11 | 11 | 11 | 11 | 11 | 11 | 11 | 11 |
| BAMS24 | 11 | 11 | 11 | 11 | 11 | 11 | 11 | 11 | 11 | 11 | 11 |
| BAMS25 | 13 | 13 | 13 | 13 | 13 | 13 | 13 | 13 | 13 | 13 | 13 |
| BAMS28 | 14 | 14 | 14 | 14 | 14 | 14 | 14 | 14 | 14 | 14 | 14 |
| BAMS30 | 68 | 68 | 68 | 72 | 72 | 72 | 72 | 79 | 72 | 72 | 79 |
| BAMS31 | 64 | 64 | 64 | 64 | 64 | 64 | 64 | 64 | 64 | 64 | 64 |
| BAMS34 | 9 | 9 | 9 | 9 | 9 | 9 | 9 | 9 | 9 | 9 | 9 |
| BAMS44 | 8 | 8 | 8 | 8 | 8 | 8 | 8 | 8 | 8 | 8 | 8 |
| BAMS51 | 9 | 9 | 9 | 9 | 9 | 9 | 9 | 9 | 9 | 9 | 9 |
| BAMS53 | 8 | 8 | 8 | 8 | 8 | 8 | 8 | 8 | 8 | 8 | 8 |
| Bavntr12 | 6 | 6 | 6 | 6 | 6 | 6 | 6 | 6 | 6 | 6 | 6 |
| Bavntr16 | 20 | 20 | 20 | 20 | 20 | 20 | 20 | 20 | 20 | 20 | 20 |
| Bavntr17 | 12 | 12 | 12 | 4 | 4 | 4 | 4 | 4 | 4 | 4 | 4 |
| Bavntr19 | 5 | 5 | 5 | 5 | 5 | 5 | 5 | 5 | 5 | 5 | 5 |
| Bavntr23 | 4 | 4 | 4 | 4 | 4 | 4 | 4 | 4 | 4 | 4 | 4 |
| Bavntr35 | 4 | 4 | 4 | 4 | 4 | 4 | 4 | 4 | 4 | 4 | 4 |
