# Supplementary material for: Epidemiological and molecular analysis of anthrax cases of the Zhambyl region Kazakhstan in 2023
Source: Front Public Health. 2025 Jul 28;13:1620930. doi: 10.3389/fpubh.2025.1620930 (PMC12336241; doi:10.3389/fpubh.2025.1620930)
Supplement: Supplementary file 4 [file Data_Sheet_1.pdf]

MLVA_bacillus (<All Characte **MLVA_bacillus**

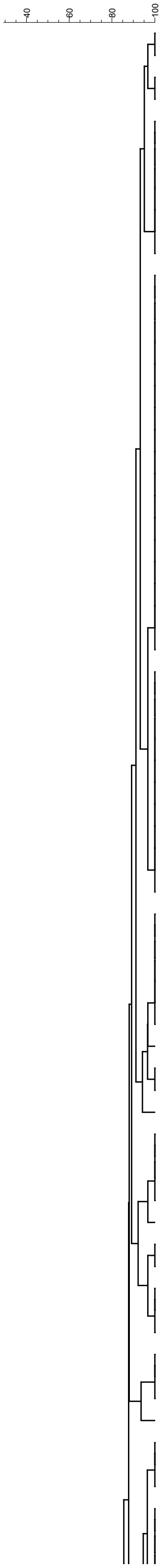

vrrA	vrrB1	vrrB2	vrrC1	vrrC2	CG3	pXO1	pXO2	BAMS01	BAMS03	BAMS05	BAMS13	BAMS15	BAMS21	BAMS22	BAMS23	BAMS24	BAMS25	BAMS28	BAMS30	BAMS31	BAMS34	BAMS44	BAMS51	BAMS53	Bavntr12	Bavntr16	Bavntr17
4	20	14	57	21	1	10	7	13	30	7	30	45	10	16	11	11	13	13	75	64	9	8	9	8	6	20	
4	20	14	57	21	1	10	7	13	30	7	30	45	10	16	11	11	13	13	75	64	9	8	9	8	6	20	
4	20	14	57	21	1	10	7	13	30	7	30	45	10	16	11	11	13	14	75	64	9	8	9	8	6	20	
4	20	14	57	21	1		7	13	30	7	30	45	10	16	11	11	13	14	75	64	9	8	9	8	6	20	
4	20	14	57	21	1	10	7	13	30	7	30	45	10	16	11	11	13	14	72	64	9	8	9	8	6	20	
4	20	14	57	21	1	10	7	13	30	7	30	45	10	16	11	11	13	14	72	64	9	8	9	8	6	20	
4	20	14	57	21	1	10	7	13	30	7	30	45	10	16	11	11	13	14	72	64	9	8	9	8	6	20	
4	20	14	57	21	1	10	7	13	30	7	30	45	10	16	11	11	13	14	72	64	9	8	9	8	6	20	
4	20	14	57	21	1	10	7	13	30	7	30	45	10	16	11	11	13	14	72	64	9	8	9	8	6	20	
4	20	14	57	21	1	10	7	13	30	7	30	45	10	16	11	11	13	14	72	64	9	8	9	8	6	20	
4	20	14	57	21	1	10	7	13	30	7	30	45	10	16	11	11	13	14	72	64	9	8	9	8	6	20	
4	20	14	57	21	1	9	7	13	30	7	30	45	10	16	11	11	13	14	79	64	9	8	9	8	6	20	
4	20	14	57	21	1	9	7	13	30	7	30	45	10	16	11	11	13	14	79	64	9	8	9	8	6	20	
4	20	14	57	21	1	9	7	13	30	7	30	45	10	16	11	11	13	14	79	64	9	8	9	8	6	20	
4	20	14	57	21	1	9	7	13	30	7	30	45	10	16	11	11	13	14	79	64	9	8	9	8	6	20	
4	20	14	57	21	1	9	7	13	30	7	30	45	10	16	11	11	13	14	79	64	9	8	9	8	6	20	
4	20	14	57	21	1	9	7	13	30	7	30	45	10	16	11	11	13	14	79	64	9	8	9	8	6	20	
4	20	14	57	21	1	9	7	13	30	7	30	45	10	16	11	11	13	14	79	64	9	8	9	8	6	20	
4	20	14	57	21	1	9	7	13	30	7	30	45	10	16	11	11	13	14	79	64	9	8	9	8	6	20	
4	20	14	57	21	1	9	7	13	30	7	30	45	10	16	11	11	13	14	79	64	9	8	9	8	6	20	
4	20	14	57	21	1	9	7	13	30	7	30	45	10	16	11	11	13	14	79	64	9	8	9	8	6	20	
4	20	14	57	21	1	9	7	13	30	7	30	45	10	16	11	11	13	14	79	64	9	8	9	8	6	20	
4	20	14	57	21	1	9	7	13	30	7	30	45	10	16	11	11	13	14	79	64	9	8	9	8	6	20	
4	20	14	57	21	1	9	7	13	30	7	30	45	10	16	11	11	13	14	79	64	9	8	9	8	6	20	
4	20	14	57	21	1	9	7	13	30	7	30	45	10	16	11	11	13	14	79	64	9	8	9	8	6	20	
4	20	14	57	21	1	9	7	13	30	7	30	45	10	16	11	11	13	14	79	64	9	8	9	8	6	20	
4	20	14	57	21	1	9	7	13	30	7	30	45	10	16	11	11	13	14	79	64	9	8	9	8	6	20	
4	20	14	57	21	1	9	7	13	30	7	30	45	10	16	11	11	13	14	79	64	9	8	9	8	6	20	
4	20	14	57	21	1	9	7	13	30	7	30	45	10	16	11	11	13	14	79	64	9	8	9	8	6	20	
4	20	14	57	21	1	8	7	13	30	7	30	45	10	16	11	11	13	14	79	64	9	8	9	8	6	20	
4	20	14	57	21	1	8	7	13	30	7	30	45	10	16	11	11	13	14	79	64	9	8	9	8	6	20	
4	20	14	57	21	1	8	7	13	30	7	30	45	10	16	11	11	13	14	79	64	9	8	9	8	6	20	
4	20	14	57	21	1	8	7	13	30	7	30	45	10	16	11	11	13	14	79	64	9	8	9	8	6	20	
4	20	14	57	21	1	8	7	13	30	7	30	45	10	16	11	11	13	14	79	64	9	8	9	8	6	20	
4	20	14	57	21	1	8	7	13	30	7	30	45	10	16	11	11	13	14	79	64	9	8	9	8	6	20	
4	20	14	57	21	1	8	7	13	30	7	30	45	10	16	11	11	13	14	79	64	9	8	9	8	6	20	
4	20	14	57	21	1	8	7	13	30	7	30	45	10	16	11	11	13	14	79	64	9	8	9	8	6	20	
4	20	14	57	21	1	8	7	13	30	7	30	45	10	16	11	11	13	14	79	64	9	8	9	8	6	20	
4	20	14	57	21	1	8	7	13	30	7	30	45	10	16	11	11	13	14	79	64	9	8	9	8	6	20	
4	20	14	57	21	1	8	7	13	30	7	30	45	10	16	11	11	13	14	79	64	9	8	9	8	6	20	
4	20	14	57	21	1	10	8	13	30	7	30	45	10	16	11	11	13	14	63	64	9	8	9	8	6	20	
4	20	14	57	21	1	10	8	13	30	7	30	45	10	16	11	11	13	14	63	64	9	8	9	8	6	20	
4	20	14	57	21	1	10	8	13	30	7	30	45	10	16	11	11	13	14	63	64	9	8	9	8	6	20	
4	20	14	57	21	1	10	8	13	30	7	30	45	10	16	11	11	13	14	63	64	9	8	9	8	6	20	
4	20	14	57	21	1	10	8	13	30	7	30	45	10	16	11	11	13	14	63	64	9	8	9	8	6	20	
4	20	14	57	21	1	10	8	13	30	7	30	45	10	16	11	11	13	14	63	64	9	8	9	8	6	20	
4	20	14	57	21	1	10	8	13	30	7	30	45	10	16	11	11	13	14	72	64	9	8	9	8	6	20	
4	20	14	57	21	1	10	8	13	30		30	45	10	16	11	11	13	14	75	64	9	8	9	8	6	20	
4	20	14	57	21	1	10	8	13	30		30	45	10	16	11	11	13	14	75	64	9	8	9	8	6	20	
4	20	14	57	21	1	9	8	13	30	7	30	45	10	16	11	11	13	14	75	64	9	8	9	8	6	20	
4	20	14	57	21	1	12	7	12	30	7	30	45	10	16	11	11	13	14	72	64	9	8	9	8	6	20	
4	20	14	57	21	1	12	7	12	30	7	30	45	10	16	11	11	13	14	72	64	9	8	9	8	6	20	
4	20	14	57	21	1	12	7	12	30	7	30	45	10	16	11	11	13	14	72	64	9	8	9	8	6	20	
4	20	14	57	21	1		7	12	30	7	30	45	10	16	11	11	13	14	72	64	9	8	9	8	6	20	
4	20	14	57	21	1	12	7	12	30	7	30	45	10	16	11	11	13	14	72	64	9	8	9	8	6	20	
4	20	14	57	21	1	11	7	12	30	7	30	45	10	16	11	8	13	14	69	64	9	8	9	8	6	20	
4	20	14	57	21	1	11	7	12	30	7	30	45	10	16	11	8	13	14	69	64	9	8	9	8	6	20	
4	20	14	57	21	1	11	7	12	30	7	30	45	10	16	11	11	13	14	69	64	9	8	9	8	6	20	
4	20	14	57	21	1	11	7	12	30	7	30	45	10	16	11	11	13	14	69	64	9	8	9	8	6	20	
4	20	14	57	21	1	11	7	12	30	7	30	45	10	16	11	11	13	14	69	64	9	8	9	8	6	20	
4	20	14	57	21	1	10	7	13	24	7	30	45	10	16	11	11	13	14	75	64	9	8	9	8	6	20	
4	20	14	57	21	1	10	7	13	24	7	30	45	10	16	11	11	13	14	75	64	9	8	9	8	6	20	
4	20	14	57	21	1	10	7	13	24	7	30	45	10	16	11	11	13	14	75	64	9	8	9	8	6	20	
4	20	14	57	21	1	8	7	13	24	7	30	45	10	16	11	11	13	14	75	64	9	8	9	8	6	20	
4	20	14	57	21	1	9		13	24	7	30	45	10	16	12	11	13	14	75	64	9	8	9	8	6		
4	20	14	57	21	1	9		13	24	7	30	45	10	16	12	11	13	14	75	64	9	8	9	8	6		
4	20	14	57	21	1	9		13	24	7	30	45	10	16	12	11	13	14	75	64	9	8	9	8	6		
4	20	14	57	21	1	9	7	13	24	7	30	45	10	16	11	11	13	14	75	64	9	8	9	8	6	20	
4	20	14	57	21	1	9	7	13	24	7	30	45	10	16	11	11	13	14	75	64	9	8	9	8	6	20	
4	20	14	57	21	1	9	7	13	24	7	30	45	10	16	11	11	13	14	75	64	9	8	9	8	6	20	

Bavntr16	Bavntr17	Bavntr19	Bavntr23	Bavntr35	Key	Region	Year	CC_MLVA31
20	4	5	4	4	KZ_Sh_2021_95	West Kazakhstan	2006	A1b_CC01
20	4	5	4	4	KZ_Sh_2021_96	Turkistan	2006	A1b_CC01
20	4	5	4	4	KZ_Sh_2021_62	Turkistan	2001	A1b_CC01
20	4	5	4	4	KZ_Sh_2021_7	Turkistan	1962	A1b_CC01
20	4	5	4	4	KZ_Sh_2021_5	Turkistan	1962	A1b_CC01
20	4	5	4	4	KZ_Sh_2021_6	Turkistan	1962	A1b_CC01
20	4	5	4	4	KZ_Sh_2021_14	Turkistan	1961	A1b_CC01
20	4	5	4	4	KZ_Sh_2021_22	Jambyl	1997	A1b_CC01
20	4	5	4	4	KZ_Sh_2021_23	Jambyl	1997	A1b_CC01
20	4	5	4	4	KZ_Sh_2021_24	Jambyl	1997	A1b_CC01
20	4	5	4	4	KZ_Sh_2021_26	Jambyl	1997	A1b_CC01
20	4	5	4	4	KZ_Sh_2021_40	Jambyl	2001	A1b_CC02
20	4	5	4	4	KZ_Sh_2021_41	Turkistan	2001	A1b_CC02
20	4	5	4	4	KZ_Sh_2021_57	Turkistan	2001	A1b_CC02
20	4	5	4	4	KZ_Sh_2021_58	Turkistan	2001	A1b_CC02
20	4	5	4	4	KZ_Sh_2021_63	Jambyl	2003	A1b_CC02
20	4	5	4	4	KZ_Sh_2021_64	Jambyl	2003	A1b_CC02
20	4	5	4	4	KZ_Sh_2021_148	Almaty	2016	A1b_CC02
20	4	5	4	4	KZ_Sh_2021_162	East Kazakhstan	2016	A1b_CC02
20	4	5	4	4	KZ_Sh_2021_163	East Kazakhstan	2016	A1b_CC02
20	4	5	4	4	KZ_Sh_2021_164	East Kazakhstan	2016	A1b_CC02
20	4	5	4	4	KZ_Sh_2021_165	East Kazakhstan	2016	A1b_CC02
20	4	5	4	4	KZ_Sh_2021_166	East Kazakhstan	2016	A1b_CC02
20	4	5	4	4	KZ_Sh_2021_167	East Kazakhstan	2016	A1b_CC02
20	4	5	4	4	KZ_Sh_2021_168	East Kazakhstan	2016	A1b_CC02
20	4	5	4	4	KZ_Sh_2021_169	East Kazakhstan	2016	A1b_CC02
20	4	5	4	4	KZ_Sh_2021_170	East Kazakhstan	2016	A1b_CC02
20	4	5	4	4	KZ_Sh_2021_171	East Kazakhstan	2016	A1b_CC02
20	4	5	4	4	KZ_Sh_2021_172	East Kazakhstan	2016	A1b_CC02
20	4	5	4	4	KZ_Sh_2021_25	Jambyl	1997	A1b_CC02
20	4	5	4	4	KZ_Sh_2021_27	Jambyl	1997	A1b_CC02
20	4	5	4	4	KZ_Sh_2021_28	Jambyl	1997	A1b_CC02
20	4	5	4	4	KZ_Sh_2021_29	Jambyl	1997	A1b_CC02
20	4	5	4	4	KZ_Sh_2021_30	Jambyl	1997	A1b_CC02
20	4	5	4	4	KZ_Sh_2021_31	Jambyl	1997	A1b_CC02
20	4	5	4	4	KZ_Sh_2021_32	Jambyl	1997	A1b_CC02
20	4	5	4	4	KZ_Sh_2021_33	Jambyl	1998	A1b_CC02
20	4	5	4	4	KZ_Sh_2021_34	Jambyl	1998	A1b_CC02
20	4	5	4	4	KZ_Sh_2021_35	Jambyl	1998	A1b_CC02
20	4	5	4	4	KZ_Sh_2021_36	Jambyl	1999	A1b_CC02
20	4	5	4	4	KZ_Sh_2021_8	Turkistan	1962	A1b_CC03
20	4	5	4	4	KZ_Sh_2021_9	Turkistan	1962	A1b_CC03
20	4	5	4	4	KZ_Sh_2021_15	Turkistan	1961	A1b_CC03
20	4	5	4	4	KZ_Sh_2021_16	Turkistan	1961	A1b_CC03
20	4	5	4	4	KZ_Sh_2021_18	Turkistan	1961	A1b_CC03
20	4	5	4	4	KZ_Sh_2021_20	Turkistan	1963	A1b_CC03
20	4	5	4	4	KZ_Sh_2021_51	Turkistan	2000	A1b_CC03
20	4	5	4	4	KZ_Sh_2021_45	East Kazakhstan	2002	A1b_CC03
20	4	5	4	4	KZ_Sh_2021_50	Turkistan	2000	A1b_CC03
20	4	5	4	4	KZ_Sh_2021_52	Turkistan	2000	A1b_CC03
20	4	5	4	4	KZ_Sh_2021_1	Almaty	1952	A1b_CC04
20	4	5	4	4	KZ_Sh_2021_2	Qyzylorda	1961	A1b_CC04
20	4	5	4	4	KZ_Sh_2021_19	Almaty	1963	A1b_CC04
20	4	5	4	4	KZ_Sh_2021_37	East Kazakhstan	2000	A1b_CC04
20	4	5	3	4	KZ_Sh_2021_71	Jambyl	2004	A1b_CC04
20	4	5	4	4	KZ_Sh_2021_93	East Kazakhstan	2005	A1b_CC04
20	4	5	4	4	KZ_Sh_2021_94	East Kazakhstan	2005	A1b_CC04
20	4	5	4	4	KZ_Sh_2021_60	East Kazakhstan	2001	A1b_CC04
20	4	5	4	4	KZ_Sh_2021_61	East Kazakhstan	2001	A1b_CC04
20	4	5	4	4	KZ_Sh_2021_92	East Kazakhstan	2005	A1b_CC04
20	4	5	3	4	KZ_Sh_2021_130	Turkistan	2011	A1b_CC06
20	4	5	3	4	KZ_Sh_2021_131	Turkistan	2011	A1b_CC06
20	4	5	3	4	KZ_Sh_2021_132	Turkistan	2011	A1b_CC06
20	4	5	4	4	KZ_Sh_2021_55	Turkistan	2000	A1b_CC06
		5	4	4	KZ_Sh_2021_65	East Kazakhstan	2004	A1b_CC05
		5	4	4	KZ_Sh_2021_76	East Kazakhstan	2004	A1b_CC05
		5	4	4	KZ_Sh_2021_77	East Kazakhstan	2004	A1b_CC05
20	5	5	4	4	KZ_Sh_2021_3	Qyzylorda	1960	A1b_CC05
20	5	5	4	4	KZ_Sh_2021_4	Atyrau	1959	A1b_CC05
20	5	5	4	4	KZ_Sh_2021_12	Turkistan	1962	A1b_CC05

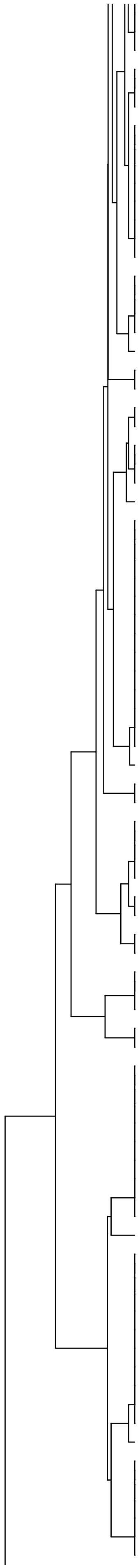

4	20	14	57	21	1	9	7	13	24	7	30	45	10	16	11	11	13	14	75	64	9	8	9	8	6	20	
4	20	14	57	21	1	9	7	13	24	7	30	45	10	16	11	11	13	14	75	64	9	8	9	8	6	20	
4	20	14	57	21	1	9	7	13	24	7	30	45	10	16	11	11	13	14	75	64	9	8	9	8	6	20	
4	20	14	57	21	1	8	7	13	30	7	30	45	10	16	11	11	13	14	75	64	9	8	9	8	6	20	
4	20	14	57	21	1	8	7	13	30	7	30	45	10	16	11	11	13	14	75	64	9	8	9	8	6	20	
4	20	14	57	21	1	8	7	13	30	7	30	45	10	16	11	11	13	14	75	64	9	8	9	8	6	20	
4	20	14	57	21	1	9	7	13	30	7	30	45	10	16	11	11	13	14	75	64	9	8	9	8	6	20	
4	20	14	57	21	1	9	7	13	30	7	30	45	10	16	11	11	13	14	75	64	9	8	9	8	6	20	
4	20	14	57	21	1	9	7	13	30	7	30	45	10	16	11	11	13	14	75	64	9	8	9	8	6	20	
4	20	14	57	21	1	9	7	13	30	7	30	45	10	16	11	11	13	14	75	64	9	8	9	8	6	20	
4	20	14	57	21	1	9	7	13	30	7	30	45	10	16	11	11	13	14	75	64	9	8	9	8	6	20	
4	20	14	57	21	1	9	7	13	30	7	30	45	10	16	11	11	13	14	75	64	9	8	9	8	6	20	
4	20	14	57	21	1	9	7	13	30	7	30	45	10	16	11	11	13	14	75	64	9	8	9	8	6	20	
4	20	14	57	21	1	9	7	13	30	7	30	45	10	16	11	11	13	14	75	64	9	8	9	8	6	20	
4	20	14	57	21	1	9	7	13	30	7	30	45	10	16	11	11	12	14	75	64	9	2	9	8	6	20	
4	20	14	57	21	1	9	7	13	30	7	30	45	10	16	11	11	12	14	75	64	9	2	9	8	6	20	
4	20	14	57	21	1	9	7	13	30	7	30	45	10	16	11	11	12	14	75	64	9	2	9	8	6	20	
4	20	14	57	21	1	9	7	13	30	7	30	45	10	16	11	11	12	14	75	64	9	2	9	8	6	20	
4	20	14	57	21	1	9	7	13	30	7	30	45	10	16	11	11	12	14	75	64	9	2	8	8	6	20	
3	20	14	57	21	1	8	7	13	29	7	30	45	10	16	11	11	13	14	69	64	9	8	9	8	6	20	
3	20	14	57	21	1	8	7	13	29	7	30	45	10	16	11	11	13	14	69	64	9	8	9	8	6	20	
5	20	14	57	21	2	10	7	13	30	7	30	38	10	14	11	11	13	14	75	64	9	8	9	8	6	20	
5	20	14	57	21	2	10	7	13	30	7	30	38	10	14	11	11	13	14	75	64	9	8	9	8	6	20	
5	20	14	57	21	2	10	7	13	30	7	30	45	10	14	11	11	13	14	75	64	9	8	9	8	6	20	
5	20	14	57	21	2	10	7	13	30	7	30	45	10	14	11	11	13	14	75	64	9	8	9	8	6	20	
5	20	14	57	21	2		7	13	30	7	30	45	10	14	11	11	13	14	75	64	9	8	9	8	6	20	
5	20	14	57	21	2	10	7	13	30	7	30	45	10	14	11	11	13	14	72	64	9	8	9	8	6	20	
4	20	14	57	21	1	10	7	13	30	7	30	45	10	14	9	11	13	14	75	64	9	8	9	8	6	20	
4	20	14	57	21	1	10	7	13	30	7	30	45	10	14	9	11	13	14	75	64	9	8	9	8	6	20	
4	20	14	57	21	1	10	7	13	30	7	30	45	10	14	9	11	13	14	75	64	9	8	9	8	6	20	
4	20	14	57	21	1	10	7	13	30	7	30	45	10	14	9	11	13	14	75	64	9	8	9	8	6	20	
4	20	14	57	21	1	10	7	13	30	7	30	45	10	14	9	11	13	14	75	64	9	8	9	8	6	20	
4	20	14	57	21	1	10	7	13	30	7	30	45	10	14	9	11	13	14	75	64	9	8	9	8	6	20	
4	20	14	57	21	1	10	7	13	30	7	30	45	10	14	9	11	13	14	75	64	9	8	9	8	6	20	
4	20	14	57	21	1	10	7	13	30	7	30	45	10	14	9	11	13	14	75	64	9	8	9	8	6	20	
4	20	14	57	21	1	10	7	13	30	7	30	45	10	14	9	11	13	14	75	64	9	8	9	8	6	20	
4	20	14	57	21	1	10	7	13	30	7	30	45	10	14	9	11	13	14	75	64	9	8	9	8	6	20	
4	20	14	57	21	1	10	7	13	30	7	30	45	10	14	9	11	13	14	75	64	9	8	9	8	6	20	
4	20	14	57	21	1		7	13	30	7	30	45	10	14	9	11	13	14	75	64	9	8	9	8	6	20	
4	20	14	57	21	1		7	13	30	7	30	45	10	14	9	11	13	14	75	64	9	8	9	8	6	20	
4	20	14	57	21	1	5	7	13	30	7	30	45	10	14	9	11	13	14	75	64	9	8	9	8	6	20	
4	20	14	57	21	1	10	7	13	30	6	27	46	10	16	11	11	13	14	75	64	9	8	9	8	6	20	
4	20	14	57	21	1	10	7	13	30	6	27	46	10	16	11	11	13	14	75	64	9	8	9	8	6	20	
4	9	3	45	21	1	8	6	13	30	7	30	45	10	16	11	11	13	14	72	64	9	8	9	8	6	20	
4	9	3	45	21	1	8	6	13	30	7	30	45	10	16	11	11	13	14	72	64	9	8	9	8	6	20	
4	9	3	45	21	1	8	6	13	30	7	30	45	10	16	11	11	13	14	72	64	9	8	9	8	6	20	
4	9	3	45	21	1	8	6	13	30	7	30	45	10	16	11	11	13	14	72	64	9	8	9	8	6	20	
4	8	3	45	21	1	8	6	13	30	7	30	45	10	16	11	11	13	14	72	64	9	8	9	8	6	20	
4	8	3	45	21	1	8	6	13	30	7	30	45	10	16	11	11	13	14	72	64	9	8	9	8	6	20	
4	9	3	45	21	1	8	7	13	30	7	30	45	10	16	11	11	13	14	79	64	9	8	9	8	6	20	
4	9	3	45	21	1	8	7	13	30	7	30	45	10	16	11	11	13	14	79	64	9	8	9	8	6	20	
4	9	3	35	17	2	8	8	13	30	6	20	46	10	16	11	11	13	14	68	64	9	8	9	8	6	20	1
4	9	3	35	17	2	8	8	13	30	6	20	46	10	16	11	11	13	14	68	64	9	8	9	8	6	20	1
4	9	3	35	17	2	8	8	13	30	6	20	46	10	16	11	11	13	14	68	64	9	8	9	8	6	20	1
4	20	14	48	21	2	8	7	13	30	6	20	46	10	16	11	11	13	14	68	64	9	8	9	8	6	20	1
4	20	14	48	21	2	8	7	13	30	6	20	46	10	16	11	11	13	14	68	64	9	8	9	8	6	20	1
4	20	14	53	17	2	7	9	16	26	7	70	24	10	16	11	11	13	14	57	64	11	8	9	8	6	20	
4	20	14	53	17	2	7	9	16	26	7	70	24	10	16	11	11	13	14	57	64	11	8	9	8	6	20	
4	20	14	53	17	2	7	9	16	26	7	70	24	10	16	11	11	13	14	57	64	11	8	9	8	6	20	
4	20	14	53	17	2	7	9	16	26	7	70	24	10	16	11	11	13	14	57	64	11	8	9	8	6	20	
4	20	14	53	17	2	7	9	16	26	7	70	24	10	16	11	11	13	14	57	64	11	8	9	8	6	20	
4	20	14	53	17	2	7	9	16	26	7	70	24	10	16	11	11	13	14	57	64	11	8	9	8	6	20	
4	20	14	53	17	2	7	9	16	26	7	70	24	10	16	11	11	13	14	57	64	11	8	9	8	6	20	
4	20	14	53	17	2	7	9	16	26	7	70	24	10	16	11	11	13	14	57	64	11	8	9	8	6	20	
4	20	14	53	17	2	7	9	16	26	7	70	24	10	16	11	11	13	14	57	64	11	8	9	8	6	20	
4	20	14	53	17	1	9	9	16	26	7	73	41	10	16	11	11	13	14	57	64	11	8	9	8	6	20	
4	20	14	53	17	2	9	9	16	28	7	73	45	10	16	11	11	13	14	57	25	11	8	9	8	6	20	
4	20	14	53	17	2	9	9	16	28	7	73	45	10	16	11	11	13	14	57	25	11	8	9	8	6	20	
4	20	14	53	17	2	9	9	16	28	7	73	45	10	16	11	11	13	14	57	25	11	8	9	8	6	20	
4	20	14	53	17	2	9	9	16	28	7	73	45	10	16	11	11	13	14	57	25	11	8	9	8	6	20	
4	20	14	53	17	2	9	9	16	28	7	73	45	10	16	11	11	13	14	57	25	11	8	9	8	6	20	
4	20	14	53	17	2	9	9	16	28	7	73	45	10	16	11	11	13	14	57	25	11	8	9	8	6	20	
4	20	14	53	17	2	9	9	16	28	7	73	45	10	16	11	11	13	14	57	25	11	8	9	8	6	20	
4	20	14	53	17	2	9	9	16	28	7	73	45	10	16	11	11	13	14	57	25	11	8	9	8	6	20	
4	20	14	53	17	2	9	9	16	28	7	73	45	10	16	11	11	13	14	57	25	11	8	9	8	6	20	
4	20	14	53	17	2	9	9	16	28	7	73	45	10	16	11	11	13	14	57	25	11	8	9	8	6	20	
4	20	14	53	17	2	9	10	16	28	7	73	45	10	16	11	11	13	14	57	25	11	8	9	8	6	20	
4	20	14	53	17	2	8	8	16	26	7	73	45	10	16	11	11	13	14	57	64	11	8	9	8	6	20	
4	20	14	53	17	2	8	8	16	26	7	73	45	10	16	11	11	13	14	57	64	11	8	9	8	6	20	
4	20	14	53	17	2	8	8	16	26	7	73	45	10	16	11	11	13	14	57	64	11	8	9	8	6	20	
4	20	14	53	17	2	8	8	16	26	7	73	45	10	16	11	11	13	14	57	64	11	8	9	8	6	20	
4	20	14	53	17	2	8	8	16	26	7	73	45	10	16	11	11	13	14	57	64	11	8	9	8	6	20	
4	20	14	53	17	2	8	8	16	26	7	73	45	10	16	11	11	13	14	57	64	11	8	9	8	6	20	

20	5	5	4	4	KZ_Sh_2021_39	.East Kazakhstan	2001	A1b_CC05
20	5	5	4	4	KZ_Sh_2021_44	.East Kazakhstan	2002	A1b_CC05
20	5	5	4	4	KZ_Sh_2021_46	.East Kazakhstan	2002	A1b_CC05
20	5	5	4	4	KZ_Sh_2021_79	.Aktobe	2004	A1b_CC05
20	5	5	4	4	KZ_Sh_2021_80	.Aktobe	2004	A1b_CC05
20	5	5	4	4	KZ_Sh_2021_81	.Aktobe	2004	A1b_CC05
20	5	5	4	4	KZ_Sh_2021_100	.West Kazakhstan	2009	A1b_CC05
20	5	5	4	4	KZ_Sh_2021_101	.West Kazakhstan	2009	A1b_CC05
20	5	5	4	4	KZ_Sh_2021_102	.West Kazakhstan	2009	A1b_CC05
20	5	5	4	4	KZ_Sh_2021_103	.West Kazakhstan	2009	A1b_CC05
20	5	5	4	4	KZ_Sh_2021_104	.West Kazakhstan	2009	A1b_CC05
20	5	5	4	4	KZ_Sh_2021_105	.West Kazakhstan	2009	A1b_CC05
20	5	5	4	4	KZ_Sh_2021_106	.West Kazakhstan	2009	A1b_CC05
20	5	5	4	4	KZ_Sh_2021_108	.West Kazakhstan	2009	A1b_CC05
20	5	5	4	4	KZ_Sh_2021_123	.Pavlodar	2010	A1b_CC11
20	5	5	4	4	KZ_Sh_2021_124	.Pavlodar	2010	A1b_CC11
20	5	5	4	4	KZ_Sh_2021_126	.Pavlodar	2010	A1b_CC11
20	5	5	4	4	KZ_Sh_2021_127	.Pavlodar	2010	A1b_CC11
20	5	5	4	4	KZ_Sh_2021_125	.Pavlodar	2010	A1b_CC11
20	4	5	4	4	KZ_Sh_2021_74	.East Kazakhstan	2004	A1b_CC07
20	4	5	4	4	KZ_Sh_2021_75	.East Kazakhstan	2004	A1b_CC07
20	4	5	4	4	KZ_Sh_2021_13	.Turkistan	1961	A1b_CC08
20	4	5	4	4	KZ_Sh_2021_17	.Turkistan	1961	A1b_CC08
20	4	5	4	4	KZ_Sh_2021_53	.Turkistan	2000	A1b_CC08
20	4	5	4	4	KZ_Sh_2021_54	.Turkistan	2000	A1b_CC08
20	4	5	4	4	KZ_Sh_2021_98	.Turkistan	2006	A1b_CC08
20	4	5	4	4	KZ_Sh_2021_59	.Turkistan	2000	A1b_CC08
20	4	5	4	4	KZ_Sh_2021_47	.West Kazakhstan	1977	A1b_CC09
20	4	5	4	4	KZ_Sh_2021_48	.West Kazakhstan	1963	A1b_CC09
20	4	5	4	4	KZ_Sh_2021_82	.West Kazakhstan	2005	A1b_CC09
20	4	5	4	4	KZ_Sh_2021_83	.West Kazakhstan	2005	A1b_CC09
20	4	5	4	4	KZ_Sh_2021_85	.West Kazakhstan	2005	A1b_CC09
20	4	5	4	4	KZ_Sh_2021_86	.West Kazakhstan	2005	A1b_CC09
20	4	5	4	4	KZ_Sh_2021_87	.West Kazakhstan	2005	A1b_CC09
20	4	5	4	4	KZ_Sh_2021_88	.West Kazakhstan	2005	A1b_CC09
20	4	5	4	4	KZ_Sh_2021_89	.West Kazakhstan	2005	A1b_CC09
20	4	5	4	4	KZ_Sh_2021_97	.Turkistan	2006	A1b_CC09
20	4	5	4	4	KZ_Sh_2021_128	.West Kazakhstan	2011	A1b_CC09
20	4	5	4	4	KZ_Sh_2021_84	.West Kazakhstan	2005	A1b_CC09
20	4	5	4	4	KZ_Sh_2021_90	.West Kazakhstan	2005	A1b_CC09
20	4	5	4	4	KZ_Sh_2021_91	.West Kazakhstan	2005	A1b_CC09
20	4	5	4	4	KZ_Sh_2021_56	.Turkistan	2000	A1b_CC10
20	4	5	4	4	KZ_Sh_2021_78	.Jambyl	2004	A1b_CC10
20	4	5	4	4	Zham_15	.		
20	4	5	4	4	Zham_18	.		
20	4	5	4	4	Zham_20	.		
20	4	5	4	4	Zham_23	.		
20	4	5	4	4	Zham_13	.		
20	4	5	4	4	Zham_14	.		
20	4	5	4	4	Zham_19	.		
20	4	5	4	4	Zham_24	.		
20	12	5	4	4	Zham_1	.		
20	12	5	4	4	Zham_11	.		
20	12	5	4	4	Zham_12	.		
20	12	5	4	4	KZ_Sh_2021_11	.Turkistan	1962	A4_CC12
20	12	5	4	4	KZ_Sh_2021_21	.Atyrau	1981	A4_CC12
20	4	4	4	5	KZ_Sh_2021_107	.West Kazakhstan	2009	A3b_CC13
20	4	4	4	5	KZ_Sh_2021_109	.West Kazakhstan	2009	A3b_CC13
20	4	4	4	5	KZ_Sh_2021_110	.West Kazakhstan	2009	A3b_CC13
20	4	4	4	5	KZ_Sh_2021_111	.West Kazakhstan	2009	A3b_CC13
20	4	4	4	5	KZ_Sh_2021_112	.West Kazakhstan	2009	A3b_CC13
20	4	4	4	5	KZ_Sh_2021_114	.West Kazakhstan	2009	A3b_CC13
20	4	4	4	5	KZ_Sh_2021_115	.West Kazakhstan	2009	A3b_CC13
20	4	4	4	5	KZ_Sh_2021_116	.West Kazakhstan	2009	A3b_CC13
20	4	4	4	5	KZ_Sh_2021_117	.West Kazakhstan	2009	A3b_CC13
20	4	4	4	5	KZ_Sh_2021_42	.	1978	A3b_CC13
20	4	4	4	5	KZ_Sh_2021_151	.Karaganda	2016	A3b_CC14
20	4	4	4	5	KZ_Sh_2021_152	.Karaganda	2016	A3b_CC14
20	4	4	4	5	KZ_Sh_2021_153	.Karaganda	2016	A3b_CC14
20	4	4	4	5	KZ_Sh_2021_154	.Karaganda	2016	A3b_CC14
20	4	4	4	5	KZ_Sh_2021_155	.Karaganda	2016	A3b_CC14
20	4	4	4	5	KZ_Sh_2021_156	.Karaganda	2016	A3b_CC14
20	4	4	4	5	KZ_Sh_2021_157	.Karaganda	2016	A3b_CC14
20	4	4	4	5	KZ_Sh_2021_158	.Karaganda	2016	A3b_CC14
20	4	4	4	5	KZ_Sh_2021_159	.Karaganda	2016	A3b_CC14
20	4	4	4	5	KZ_Sh_2021_160	.Karaganda	2016	A3b_CC14
20	4	4	4	5	KZ_Sh_2021_150	.Karaganda	2016	A3b_CC14
20	4	4	4	5	KZ_Sh_2021_66	.East Kazakhstan	2004	A3b_CC15
20	4	4	4	5	KZ_Sh_2021_67	.East Kazakhstan	2004	A3b_CC15
20	4	4	4	5	KZ_Sh_2021_68	.East Kazakhstan	2004	A3b_CC15
20	4	4	4	5	KZ_Sh_2021_69	.East Kazakhstan	2004	A3b_CC15
20	4	4	4	5	KZ_Sh_2021_72	.East Kazakhstan	2004	A3b_CC15
20	4	4	4	5	KZ_Sh_2021_73	.East Kazakhstan	2004	A3b_CC15

3	23	14	53	17	2	9	7	14	27	7	24	45	10	15	10	11	13	11	17	6		8	6	6	5	21
3	23	14	53	17	2	9	7	14	27	7	24	45	10	15	10	11	13	11	17	6		8	6	6	5	21
3	23	14	53	17	2	9	7	14	27	7	24	45	10	15	10	11	13	11	17	6		8	6	6	5	21
3	23	14	53	17	2	9	7	14	27	7	24	45	10	15	10	11	13	11	17	6		8	6	6	5	21
3	23	14	53	17	2	9	7	14	27	7	24	45	10	15	10	11	13	11	17	6		8	6	6	5	21
3	23	14	53	17	2	9	7	14	27	7	24	45	10	15	10	11	13	11	17	6		8	6	6	5	21
3	23	14	53	17	2	9	7	14	27	7	24	45	10	15	10	11	13	11	17	6		8	6	6	5	21
3	23	14	53	17	2	9	7	14	27	7	24	45	10	15	10	11	13	11	17	6		8	6	6	5	21
3	23	14	53	17	2	9	7	14	27	7	24	45	10	15	10	11	13	11	17	6		8	6	6	5	21
3	23	14	53	17	2	9	7	14	27	7	24	45	10	15	10	11	13	11	17	6		8	6	6	5	21
3	23	14	53	17	2	9	7	14	27	7	24	45	10	15	10	11	13	11	17	6		8	6	6	5	21
3	23	14	53	17	2	9	7	14	27	7	24	45	10	15	10	11	13	11	17	6		8	6	6	5	21

21	3	4	3	5	KZ_Sh_2021_173	.Pavlodar	2016	B1_CC16
21	3	4	3	5	KZ_Sh_2021_174	.Pavlodar	2016	B1_CC16
21	3	4	3	5	KZ_Sh_2021_175	.Pavlodar	2016	B1_CC16
21	3	4	3	5	KZ_Sh_2021_176	.Pavlodar	2016	B1_CC16
21	3	4	3	5	KZ_Sh_2021_177	.Pavlodar	2016	B1_CC16
21	3	4	3	5	KZ_Sh_2021_178	.Pavlodar	2016	B1_CC16
21	3	4	3	5	KZ_Sh_2021_179	.Pavlodar	2016	B1_CC16
21	3	4	3	5	KZ_Sh_2021_180	.Pavlodar	2016	B1_CC16
21	3	4	3	5	KZ_Sh_2021_181	.Pavlodar	2016	B1_CC16
21	3	4	3	5	KZ_Sh_2021_182	.Pavlodar	2016	B1_CC16
21	3	4	3	5	KZ_Sh_2021_183	.Pavlodar	2016	B1_CC16
21	3	4	3	5	KZ_Sh_2021_184	.Pavlodar	2016	B1_CC16
