# Supplementary material for: Epidemiological and molecular analysis of anthrax cases of the Zhambyl region Kazakhstan in 2023
Source: Front Public Health. 2025 Jul 28;13:1620930. doi: 10.3389/fpubh.2025.1620930 (PMC12336241; doi:10.3389/fpubh.2025.1620930)
Supplement: Supplementary file 5 [file Image_1.pdf]

Supplementary Figure 1. Phylogenetic comparison of MLVA-31 genotypes from *B. anthracis* isolates in Zhambyl region 2023 with previously published genotypes from Kazakhstan

MLVA_bacillus (<All Characte **MLVA_bacillus**

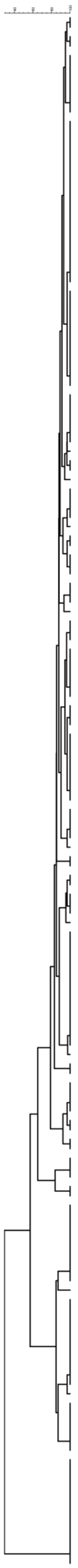

vrrA	vrrB1	vrrB2	vrrC1	vrrC2	CG3	pXO1	pXO2	BAMS01	BAMS03	BAMS05	BAMS13	BAMS15	BAMS21	BAMS22	BAMS23	BAMS24	BAMS25	BAMS28	BAMS30	BAMS31	BAMS34	BAMS44	BAMS51	BAMS53	Bavntr12	Bavntr16	Bavntr17	Bavntr19	Bavntr23	Bavntr35	Key	Region	Year	CC_MLVA31
4	20	14	57	21	1	10	7	13	30	7	30	45	10	16	11	11	13	13	75	64	9	8	9	8	6	20	4	5	4	4	KZ_Sh_2021_95	West Kazakhstan	2006	A1b_CC01
4	20	14	57	21	1	10	7	13	30	7	30	45	10	16	11	11	13	13	75	64	9	8	9	8	6	20	4	5	4	4	KZ_Sh_2021_96	Turkistan	2006	A1b_CC01
4	20	14	57	21	1	10	7	13	30	7	30	45	10	16	11	11	13	14	75	64	9	8	9	8	6	20	4	5	4	4	KZ_Sh_2021_62	Turkistan	2001	A1b_CC01
4	20	14	57	21	1		7	13	30	7	30	45	10	16	11	11	13	14	75	64	9	8	9	8	6	20	4	5	4	4	KZ_Sh_2021_7	Turkistan	1962	A1b_CC01
4	20	14	57	21	1	10	7	13	30	7	30	45	10	16	11	11	13	14	72	64	9	8	9	8	6	20	4	5	4	4	KZ_Sh_2021_5	Turkistan	1962	A1b_CC01
4	20	14	57	21	1	10	7	13	30	7	30	45	10	16	11	11	13	14	72	64	9	8	9	8	6	20	4	5	4	4	KZ_Sh_2021_6	Turkistan	1962	A1b_CC01
4	20	14	57	21	1	10	7	13	30	7	30	45	10	16	11	11	13	14	72	64	9	8	9	8	6	20	4	5	4	4	KZ_Sh_2021_14	Turkistan	1961	A1b_CC01
4	20	14	57	21	1	10	7	13	30	7	30	45	10	16	11	11	13	14	72	64	9	8	9	8	6	20	4	5	4	4	KZ_Sh_2021_22	Jambyl	1997	A1b_CC01
4	20	14	57	21	1	10	7	13	30	7	30	45	10	16	11	11	13	14	72	64	9	8	9	8	6	20	4	5	4	4	KZ_Sh_2021_23	Jambyl	1997	A1b_CC01
4	20	14	57	21	1	10	7	13	30	7	30	45	10	16	11	11	13	14	72	64	9	8	9	8	6	20	4	5	4	4	KZ_Sh_2021_24	Jambyl	1997	A1b_CC01
4	20	14	57	21	1	10	7	13	30	7	30	45	10	16	11	11	13	14	72	64	9	8	9	8	6	20	4	5	4	4	KZ_Sh_2021_26	Jambyl	1997	A1b_CC01
4	20	14	57	21	1	9	7	13	30	7	30	45	10	16	11	11	13	14	79	64	9	8	9	8	6	20	4	5	4	4	KZ_Sh_2021_40	Jambyl	2001	A1b_CC02
4	20	14	57	21	1	9	7	13	30	7	30	45	10	16	11	11	13	14	79	64	9	8	9	8	6	20	4	5	4	4	KZ_Sh_2021_41	Turkistan	2001	A1b_CC02
4	20	14	57	21	1	9	7	13	30	7	30	45	10	16	11	11	13	14	79	64	9	8	9	8	6	20	4	5	4	4	KZ_Sh_2021_57	Turkistan	2001	A1b_CC02
4	20	14	57	21	1	9	7	13	30	7	30	45	10	16	11	11	13	14	79	64	9	8	9	8	6	20	4	5	4	4	KZ_Sh_2021_58	Turkistan	2001	A1b_CC02
4	20	14	57	21	1	9	7	13	30	7	30	45	10	16	11	11	13	14	79	64	9	8	9	8	6	20	4	5	4	4	KZ_Sh_2021_63	Jambyl	2003	A1b_CC02
4	20	14	57	21	1	9	7	13	30	7	30	45	10	16	11	11	13	14	79	64	9	8	9	8	6	20	4	5	4	4	KZ_Sh_2021_64	Jambyl	2003	A1b_CC02
4	20	14	57	21	1	9	7	13	30	7	30	45	10	16	11	11	13	14	79	64	9	8	9	8	6	20	4	5	4	4	KZ_Sh_2021_148	Almaty	2016	A1b_CC02
4	20	14	57	21	1	9	7	13	30	7	30	45	10	16	11	11	13	14	79	64	9	8	9	8	6	20	4	5	4	4	KZ_Sh_2021_162	East Kazakhstan	2016	A1b_CC02
4	20	14	57	21	1	9	7	13	30	7	30	45	10	16	11	11	13	14	79	64	9	8	9	8	6	20	4	5	4	4	KZ_Sh_2021_163	East Kazakhstan	2016	A1b_CC02
4	20	14	57	21	1	9	7	13	30	7	30	45	10	16	11	11	13	14	79	64	9	8	9	8	6	20	4	5	4	4	KZ_Sh_2021_164	East Kazakhstan	2016	A1b_CC02
4	20	14	57	21	1	9	7	13	30	7	30	45	10	16	11	11	13	14	79	64	9	8	9	8	6	20	4	5	4	4	KZ_Sh_2021_165	East Kazakhstan	2016	A1b_CC02
4	20	14	57	21	1	9	7	13	30	7	30	45	10	16	11	11	13	14	79	64	9	8	9	8	6	20	4	5	4	4	KZ_Sh_2021_166	East Kazakhstan	2016	A1b_CC02
4	20	14	57	21	1	9	7	13	30	7	30	45	10	16	11	11	13	14	79	64	9	8	9	8	6	20	4	5	4	4	KZ_Sh_2021_167	East Kazakhstan	2016	A1b_CC02
4	20	14	57	21	1	9	7	13	30	7	30	45	10	16	11	11	13	14	79	64	9	8	9	8	6	20	4	5	4	4	KZ_Sh_2021_168	East Kazakhstan	2016	A1b_CC02
4	20	14	57	21	1	9	7	13	30	7	30	45	10	16	11	11	13	14	79	64	9	8	9	8	6	20	4	5	4	4	KZ_Sh_2021_169	East Kazakhstan	2016	A1b_CC02
4	20	14	57	21	1	9	7	13	30	7	30	45	10	16	11	11	13	14	79	64	9	8	9	8	6	20	4	5	4	4	KZ_Sh_2021_170	East Kazakhstan	2016	A1b_CC02
4	20	14	57	21	1	9	7	13	30	7	30	45	10	16	11	11	13	14	79	64	9	8	9	8	6	20	4	5	4	4	KZ_Sh_2021_171	East Kazakhstan	2016	A1b_CC02
4	20	14	57	21	1	9	7	13	30	7	30	45	10	16	11	11	13	14	79	64	9	8	9	8	6	20	4	5	4	4	KZ_Sh_2021_172	East Kazakhstan	2016	A1b_CC02
4	20	14	57	21	1	8	7	13	30	7	30	45	10	16	11	11	13	14	79	64	9	8	9	8	6	20	4	5	4	4	KZ_Sh_2021_25	Jambyl	1997	A1b_CC02
4	20	14	57	21	1	8	7	13	30	7	30	45	10	16	11	11	13	14	79	64	9	8	9	8	6	20	4	5	4	4	KZ_Sh_2021_27	Jambyl	1997	A1b_CC02
4	20	14	57	21	1	8	7	13	30	7	30	45	10	16	11	11	13	14	79	64	9	8	9	8	6	20	4	5	4	4	KZ_Sh_2021_28	Jambyl	1997	A1b_CC02
4	20	14	57	21	1	8	7	13	30	7	30	45	10	16	11	11	13	14	79	64	9	8	9	8	6	20	4	5	4	4	KZ_Sh_2021_29	Jambyl	1997	A1b_CC02
4	20	14	57	21	1	8	7	13	30	7	30	45	10	16	11	11	13	14	79	64	9	8	9	8	6	20	4	5	4	4	KZ_Sh_2021_30	Jambyl	1997	A1b_CC02
4	20	14	57	21	1	8	7	13	30	7	30	45	10	16	11	11	13	14	79	64	9	8	9	8	6	20	4	5	4	4	KZ_Sh_2021_31	Jambyl	1997	A1b_CC02
4	20	14	57	21	1	8	7	13	30	7	30	45	10	16	11	11	13	14	79	64	9	8	9	8	6	20	4	5	4	4	KZ_Sh_2021_32	Jambyl	1997	A1b_CC02
4	20	14	57	21	1	8	7	13	30	7	30	45	10	16	11	11	13	14	79	64	9	8	9	8	6	20	4	5	4	4	KZ_Sh_2021_33	Jambyl	1998	A1b_CC02
4	20	14	57	21	1	8	7	13	30	7	30	45	10	16	11	11	13	14	79	64	9	8	9	8	6	20	4	5	4	4	KZ_Sh_2021_34	Jambyl	1998	A1b_CC02
4	20	14	57	21	1	8	7	13	30	7	30	45	10	16	11	11	13	14	79	64	9	8	9	8	6	20	4	5	4	4	KZ_Sh_2021_35	Jambyl	1998	A1b_CC02
4	20	14	57	21	1	8	7	13	30	7	30	45	10	16	11	11	13	14	79	64	9	8	9	8	6	20	4	5	4	4	KZ_Sh_2021_36	Jambyl	1999	A1b_CC02
4	20	14	57	21	1	10	8	13	30	7	30	45	10	16	11	11	13	14	63	64	9	8	9	8	6	20	4	5	4	4	KZ_Sh_2021_8	Turkistan	1962	A1b_CC03
4	20	14	57	21	1	10	8	13	30	7	30	45	10	16	11	11	13	14	63	64	9	8	9	8	6	20	4	5	4	4	KZ_Sh_2021_9	Turkistan	1962	A1b_CC03
4	20	14	57	21	1	10	8	13	30	7	30	45	10	16	11	11	13	14	63	64	9	8	9	8	6	20	4	5	4	4	KZ_Sh_2021_15	Turkistan	1961	A1b_CC03
4	20	14	57	21	1	10	8	13	30	7	30	45	10	16	11	11	13	14	63	64	9	8	9	8	6	20	4	5	4	4	KZ_Sh_2021_16	Turkistan	1961	A1b_CC03
4	20	14	57	21	1	10	8	13	30	7	30	45	10	16	11	11	13	14	63	64	9	8	9	8	6	20	4	5	4	4	KZ_Sh_2021_18	Turkistan	1961	A1b_CC03
4	20	14	57	21	1	10	8	13	30	7	30	45	10	16	11	11	13	14	63	64	9	8	9	8	6	20	4	5	4	4	KZ_Sh_2021_20	Turkistan	1963	A1b_CC03
4	20	14	57	21	1	10	8	13	30	7	30	45	10	16	11	11	13	14	72	64	9	8	9	8	6	20	4	5	4	4	KZ_Sh_2021_51	Turkistan	2000	A1b_CC03
4	20	14	57	21	1	10	8	13	30		30	45	10	16	11	11	13	14	75	64	9	8	9	8	6	20	4	5	4	4	KZ_Sh_2021_45	East Kazakhstan	2002	A1b_CC03
4	20	14	57	21	1	10	8	13	30		30	45	10	16	11	11	13	14	75	64	9	8	9	8	6	20	4	5	4	4	KZ_Sh_2021_50	Turkistan	2000	A1b_CC03
4	20	14	57	21	1	9	8	13	30	7	30	45	10	16	11	11	13	14	75	64	9	8	9	8	6	20	4	5	4	4	KZ_Sh_2021_52	Turkistan	2000	A1b_CC03
4	20	14	57	21	1	12	7	12	30	7	30	45	10	16	11	11	13	14	72	64	9	8	9	8	6	20	4	5	4	4	KZ_Sh_2021_1	Almaty	1952	A1b_CC04
4	20	14	57	21	1	12	7	12	30	7	30	45	10	16	11	11	13	14	72	64	9	8	9	8	6	20	4	5	4	4	KZ_Sh_2021_2	Qyzylorda	1961	A1b_CC04
4	20	14	57	21	1	12	7	12	30	7	30	45	10	16	11	11	13	14	72	64	9	8	9	8	6	20	4	5	4	4	KZ_Sh_2021_19	Almaty	1963	A1b_CC04
4	20	14	57	21	1		7	12	30	7	30	45	10	16	11	11	13	14	72	64	9	8	9	8	6	20	4	5	4	4	KZ_Sh_2021_37	East Kazakhstan	2000	A1b_CC04
4	20	14	57	21	1	12	7	12	30	7	30	45	10	16	11	11	13	14	72	64	9	8	9	8	6	20	4	5	3	4	KZ_Sh_2021_71	Jambyl	2004	A1b_CC04
4	20	14	57	21	1	11	7	12	30	7	30	45	10	16	11	8	13	14	69	64	9	8	9	8	6	20	4	5	4	4	KZ_Sh_2021_93	East Kazakhstan	2005	A1b_CC04
4	20	14	57	21	1	11	7	12	30	7	30	45	10	16	11	8	13	14	69	64	9	8	9	8	6	20	4	5	4	4	KZ_Sh_2021_94	East Kazakhstan	2005	A1b_CC04
4	20	14	57	21	1	11	7	12	30	7	30	45	10	16	11	11	13	14	69	64	9	8	9	8	6	20	4	5	4	4	KZ_Sh_2021_60	East Kazakhstan	2001	A1b_CC04
4	20	14	57	21	1	11	7	12	30	7	30	45	10	16	11	11	13	14	69	64	9	8	9	8	6	20	4	5	4	4	KZ_Sh_2021_61	East Kazakhstan	2001	A1b_CC04
4	20	14	57	21	1	11	7	12	30	7	30	45	10	16	11	11	13	14	69	64	9	8	9	8	6	20	4	5	4	4	KZ_Sh_2021_92	East Kazakhstan	2005	A1b_CC04
4	20	14	57	21	1	10	7	13	24	7	30	45	10	16	11	11	13	14	75	64	9	8	9	8	6	20	4	5	3	4	KZ_Sh_2021_130	Turkistan	2011	A1b_CC06
4	20	14	57	21	1	10	7	13	24	7	30	45	10	16	11	11	13	14	75	64	9	8	9	8	6	20	4	5	3	4	KZ_Sh_2021_131	Turkistan	2011	A1b_CC06
4	20	14	57	21	1	10	7	13	24	7	30	45	10	16	11	11	13	14	75	64	9	8	9	8	6	20	4	5	3	4	KZ_Sh_2021_132	Turkistan	2011	A1b_CC06
4	20	14	57	21	1	8	7	13	24	7	30	45	10	16	11	11	13	14	75	64	9	8	9	8	6	20	4	5	4	4	KZ_Sh_2021_55	Turkistan	2000	A1b_CC06
4	20	14	57	21	1	9		13	24	7	30	45	10	16	12	11	13	14	75	64	9	8	9	8	6			5	4	4	KZ_Sh_2021_65	East Kazakhstan	2004	A1b_CC05
4	20	14	57	21	1	9		13	24	7	30	45	10	16	12	11	13	14	75	64	9	8	9	8	6			5	4	4	KZ_Sh_2021_76	East Kazakhstan	2004	A1b_CC05
4	20	14	57	21	1	9		13	24	7	30	45	10	16	12	11	13	14	75	64	9	8	9	8	6			5	4	4	KZ_Sh_2021_77	East Kazakhstan	2004	A1b_CC05
4	20	14	57	21	1	9	7	13	24	7	30	45	10	16	11	11	13	14	75	64	9	8	9	8	6	20	5	5	4	4	KZ_Sh_2021_3	Qyzylorda	1960	A1b_CC05
4	20	14	57	21	1	9	7	13	24	7	30	45	10	16	11	11	13	14	75	64	9	8	9	8	6	20	5	5	4	4	KZ_Sh_2021_4	Atyrau	1959	A1b_CC05
4	20	14	57	21	1	9	7	13	24	7	30	45	10	16	11	11	13	14	75	64	9	8	9	8	6	20	5	5	4	4	KZ_Sh_2021_12	Turkistan	1962	A1b_CC05
4	20	14	57	21	1	9	7	13	24	7	30	45	10	16	11	11	13	14	75	64	9	8	9	8	6	20	5	5	4	4	KZ_Sh_2021_39	East Kazakhstan	2001	A1b_CC05
4	20	14	57	21	1	9	7	13	24	7	30	45	10	16	11	11	13	14	75	64	9	8	9	8	6	20	5	5	4	4	KZ_Sh_2021_44	East Kazakhstan	2002	A1b_CC05
4	20	14	57	21	1	9	7	13	24	7	30	45	10	16	11	11	13	14	75	64	9	8	9	8	6	20	5	5	4	4	KZ_Sh_2021_46	East Kazakhstan	2002	A1b_CC05
4	20	14	57	21	1	8	7	13	30	7	30	45	10	16	11	11	13	14	75	64	9	8	9	8	6	20	5	5	4	4	KZ_Sh_2021_79	Aktobe	2004	A1b_CC05
4	20	14	57	21	1	8	7	13	30	7	30	45	10	16	11	11	13	14	75	64	9	8	9	8	6	20	5	5	4	4	KZ_Sh_2021_80	Aktobe	2004	A1b_CC05
4	20	14	57	21	1	8	7	13	30	7	30	45	10	16	11	11	13	14	75	64	9	8	9	8	6	20	5	5	4	4	KZ_Sh_2021_81	Aktobe	2004	A1b_CC05
4	20	14	57	21	1	9	7	13	30	7	30	45	10	16	11	11	13	14	75	64	9	8	9	8	6	20	5	5	4	4	KZ_Sh_2021_100	West Kazakhstan	2009	A1b_CC05
4	20	14	57	21	1	9	7	13	30	7	30	45	10	16	11	11	13	14	75	64	9	8	9	8	6	20	5	5	4	4	KZ_Sh_2021_101	West Kazakhstan	2009	A1b_CC05
4	20	14	57	21	1	9	7	13	30	7	30	45	10	16	11	11	13	14	75	64	9	8	9	8	6	20	5	5	4	4	KZ_Sh_2021_102	West Kazakhstan	2009	A1b_CC05
4	20	14	57	21	1	9	7	13	30	7	30	45	10	16	11	11	13	14	75	64	9	8	9	8	6	20	5	5	4	4	KZ_Sh_2021_103	West Kazakhstan	2009	A1b_CC05
4	20	14	57	21	1	9	7	13	30	7	30	45	10	16	11	11	13	14	75	64	9	8	9	8	6	20	5	5	4	4	KZ_Sh_2021_104	West Kazakhstan	2009	A1b_CC05
4	20	14	57	21	1	9	7	13	30	7	30	45	10	16	11	11	13	14	75	64	9	8	9	8	6	20	5	5	4	4	KZ_Sh_2021_105	West Kazakhstan	2009	A1b_CC05
4	20	14	57	21	1	9	7	13	30	7	30	45	10	16	11	11	13	14	75	64	9	8	9	8	6	20	5	5	4	4	KZ_Sh_2021_106	West Kazakhstan	2009	A1b_CC05
4	20	14	57	21	1	9	7	13	30	7	30	45	10	16	11	11	13	14	75	64	9	8	9	8	6	20	5	5	4	4	KZ_Sh_2021_108	West Kazakhstan	2009	A1b_CC05
4	20	14	57	21	1	9	7	13	30	7	30	45	10	16	11	11	12	14	75	64	9	2	9	8	6	20	5	5	4	4	KZ_Sh_2021_123	Pavlodar	2010	A1b_CC11
4	20	14	57	21	1	9	7	13	30	7	30	45	10	16	11	11	12	14	75	64	9	2	9	8	6	20	5	5	4	4	KZ_Sh_2021_124	Pavlodar	2010	A1b_CC11
4	20	14	57	21	1	9	7	13	30	7	30	45	10	16	11	11	12	14	75	64	9	2	9	8	6	20	5	5	4	4	KZ_Sh_2021_126	Pavlodar	2010	A1b_CC11
4	20	14	57	21	1	9	7	13	30	7	30	45	10	16	11	11	12	14	75	64	9	2	9	8	6	20	5	5	4	4	KZ_Sh_2021_127	Pavlodar	2010	A1b_CC11
4	20	14	57	21	1	9	7	13	30	7	30	45	10	16	11	11	12	14	75	64	9	2	8	8	6	20	5	5	4	4	KZ_Sh_2021_125	Pavlodar	2010	A1b_CC11
3	20	14	57	21	1	8	7	13	29	7	30	45	10	16	11	11	13	14	69	64	9	8	9	8	6	20	4	5	4	4	KZ_Sh_2021_74	East Kazakhstan	2004	A1b_CC07
3	20	14	57	21	1	8	7	13	29	7	30	45	10	16	11	11	13	14	69	64	9	8	9	8	6	20	4	5	4	4	KZ_Sh_2021_75	East Kazakhstan	2004	A1b_CC07
5	20	14	57	21	2	10	7	13	30	7	30	38	10	14	11	11	13	14	75	64	9	8	9	8	6	20	4	5	4	4	KZ_Sh_2021_13	Turkistan	1961	A1b_CC08
5	20	14	57	21	2	10	7	13	30	7	30	38	10	14	11	11	13	14	75	64	9	8	9	8	6	20	4	5	4	4	KZ_Sh_2021_17	Turkistan	1961	A1b_CC08
5	20	14	57	21	2	10	7	13	30	7	30	45	10	14	11	11	13	14	75	64	9	8	9	8	6	20	4	5	4	4	KZ_Sh_2021_53	Turkistan	2000	A1b_CC08
5	20	14	57	21	2	10	7	13	30	7	30	45	10	14	11	11	13	14	75	64	9	8	9	8	6	20	4	5	4	4	KZ_Sh_2021_54	Turkistan	2000	A1b_CC08
5	20	14	57	21	2		7	13	30	7	30	45	10	14	11	11	13	14	75	64	9	8	9	8	6	20	4	5	4	4	KZ_Sh_2021_98	Turkistan	2006	A1b_CC08
5	20	14	57	21	2	10	7	13	30	7	30	45	10	14	11	11	13	14	72	64	9	8	9	8	6	20	4	5	4	4	KZ_Sh_2021_59	Turkistan	2000	A1b_CC08
4	20	14	57	21	1	10	7	13	30	7	30	45	10	14	9	11	13	14	75	64	9	8	9	8	6	20	4	5	4	4	KZ_Sh_2021_47	West Kazakhstan	1977	A1b_CC09
4	20	14	57	21	1	10	7	13	30	7	30	45	10	14	9	11	13	14	75	64	9	8	9	8	6	20	4	5	4	4	KZ_Sh_2021_48	West Kazakhstan	1963	A1b_CC09
4	20	14	57	21	1	10	7	13	30	7	30	45	10	14	9	11	13	14	75	64	9	8	9	8	6	20	4	5	4	4	KZ_Sh_2021_82	West Kazakhstan	2005	A1b_CC09
4	20	14	57	21	1	10	7	13	30	7	30	45	10	14	9	11	13	14	75	64	9	8	9	8	6	20	4	5	4	4	KZ_Sh_2021_83	West Kazakhstan	2005	A1b_CC09
4	20	14	57	21	1	10	7	13	30	7	30	45	10	14	9	11	13	14	75	64	9	8	9	8	6	20	4	5	4	4	KZ_Sh_2021_85	West Kazakhstan	2005	A1b_CC09
4	20	14	57	21	1	10	7	13	30	7	30	45	10	14	9	11	13	14	75	64	9	8	9	8	6	20	4	5	4	4	KZ_Sh_2021_86	West Kazakhstan	2005	A1b_CC09
4	20	14	57	21	1	10	7	13	30	7	30	45	10	14	9	11	13	14	75	64	9	8	9	8	6	20	4	5	4	4	KZ_Sh_2021_87	West Kazakhstan	2005	A1b_CC09
4	20	14	57	21	1	10	7	13	30	7	30	45	10	14	9	11	13	14	75	64	9	8	9	8	6	20	4	5	4	4	KZ_Sh_2021_88	West Kazakhstan	2005	A1b_CC09
4	20	14	57	21	1	10	7	13	30	7	30	45	10	14	9	11	13	14	75	64	9	8	9	8	6	20	4	5	4	4	KZ_Sh_2021_89	West Kazakhstan	2005	A1b_CC09
4	20	14	57	21	1	10	7	13	30	7	30	45	10	14	9	11	13	14	75	64	9	8	9	8	6	20	4	5	4	4	KZ_Sh_2021_97	Turkistan	2006	A1b_CC09
4	20	14	57	21	1	10	7	13	30	7	30	45	10	14	9	11	13	14	75	64	9	8	9	8	6	20	4	5	4	4	KZ_Sh_2021_128	West Kazakhstan	2011	A1b_CC09
4	20	14	57	21	1		7	13	30	7	30	45	10	14	9	11	13	14	75	64	9	8	9	8	6	20	4	5	4	4	KZ_Sh_2021_84	West Kazakhstan	2005	A1b_CC09
4	20	14	57	21	1		7	13	30	7	30	45	10	14	9	11	13	14	75	64	9	8	9	8	6	20	4	5	4	4	KZ_Sh_2021_90	West Kazakhstan	2005	A1b_CC09
4	20	14	57	21	1	5	7	13	30	7	30	45	10	14	9	11	13	14	75	64	9	8	9	8	6	20	4	5	4	4	KZ_Sh_2021_91	West Kazakhstan	2005	A1b_CC09
4	20	14	57	21	1	10	7	13	30	6	27	46	10	16	11	11	13	14	75	64	9	8	9	8	6	20	4	5	4	4	KZ_Sh_2021_56	Turkistan	2000	A1b_CC10
4	20	14	57	21	1	10	7	13	30	6	27	46	10	16	11	11	13	14	75	64	9	8	9	8	6	20	4	5	4	4	KZ_Sh_2021_78	Jambyl	2004	A1b_CC10
4	9	3	45	21	1	8	6	13	30	7	30	45	10	16	11	11	13	14	72	64	9	8	9	8	6	20	4	5	4	4	Zham_15			
4	9	3	45	21	1	8	6	13	30	7	30	45	10	16	11	11	13	14	72	64	9	8	9	8	6	20	4	5	4	4	Zham_18			
4	9	3	45	21	1	8	6	13	30	7	30	45	10	16	11	11	13	14	72	64	9	8	9	8	6	20	4	5	4	4	Zham_20			
4	9	3	45	21	1	8	6	13	30	7	30	45	10	16	11	11	13	14	72	64	9	8	9	8	6	20	4	5	4	4	Zham_23			
4	8	3	45	21	1	8	6	13	30	7	30	45	10	16	11	11	13	14	72	64	9	8	9	8	6	20	4	5	4	4	Zham_13			
4	8	3	45	21	1	8	6	13	30	7	30	45	10	16	11	11	13	14	72	64	9	8	9	8	6	20	4	5	4	4	Zham_14			
4	9	3	45	21	1	8	7	13	30	7	30	45	10	16	11	11	13	14	79	64	9	8	9	8	6	20	4	5	4	4	Zham_19			
4	9	3	45	21	1	8	7	13	30	7	30	45	10	16	11	11	13	14	79	64	9	8	9	8	6	20	4	5	4	4	Zham_24			
4	9	3	35	17	2	8	8	13	30	6	20	46	10	16	11	11	13	14	68	64	9	8	9	8	6	20	12	5	4	4	Zham_1			
4	9	3	35	17	2	8	8	13	30	6	20	46	10	16	11	11	13	14	68	64	9	8	9	8	6	20	12	5	4	4	Zham_11			
4	9	3	35	17	2	8	8	13	30	6	20	46	10	16	11	11	13	14	68	64	9	8	9	8	6	20	12	5	4	4	Zham_12			
4	20	14	48	21	2	8	7	13	30	6	20	46	10	16	11	11	13	14	68	64	9	8	9	8	6	20	12	5	4	4	KZ_Sh_2021_11	Turkistan	1962	A4_CC12
4	20	14	48	21	2	8	7	13	30	6	20	46	10	16	11	11	13	14	68	64	9	8	9	8	6	20	12	5	4	4	KZ_Sh_2021_21	Atyrau	1981	A4_CC12
4	20	14	53	17	2	7	9	16	26	7	70	24	10	16	11	11	13	14	57	64	11	8	9	8	6	20	4	4	4	5	KZ_Sh_2021_107	West Kazakhstan	2009	A3b_CC13
4	20	14	53	17	2	7	9	16	26	7	70	24	10	16	11	11	13	14	57	64	11	8	9	8	6	20	4	4	4	5	KZ_Sh_2021_109	West Kazakhstan	2009	A3b_CC13
4	20	14	53	17	2	7	9	16	26	7	70	24	10	16	11	11	13	14	57	64	11	8	9	8	6	20	4	4	4	5	KZ_Sh_2021_110	West Kazakhstan	2009	A3b_CC13
4	20	14	53	17	2	7	9	16	26	7	70	24	10	16	11	11	13	14	57	64	11	8	9	8	6	20	4	4	4	5	KZ_Sh_2021_111	West Kazakhstan	2009	A3b_CC13
4	20	14	53	17	2	7	9	16	26	7	70	24	10	16	11	11	13	14	57	64	11	8	9	8	6	20	4	4	4	5	KZ_Sh_2021_112	West Kazakhstan	2009	A3b_CC13
4	20	14	53	17	2	7	9	16	26	7	70	24	10	16	11	11	13	14	57	64	11	8	9	8	6	20	4	4	4	5	KZ_Sh_2021_114	West Kazakhstan	2009	A3b_CC13
4	20	14	53	17	2	7	9	16	26	7	70	24	10	16	11	11	13	14	57	64	11	8	9	8	6	20	4	4	4	5	KZ_Sh_2021_115	West Kazakhstan	2009	A3b_CC13
4	20	14	53	17	2	7	9	16	26	7	70	24	10	16	11	11	13	14	57	64	11	8	9	8	6	20	4	4	4	5	KZ_Sh_2021_116	West Kazakhstan	2009	A3b_CC13
4	20	14	53	17	2	7	9	16	26	7	70	24	10	16	11	11	13	14	57	64	11	8	9	8	6	20	4	4	4	5	KZ_Sh_2021_117	West Kazakhstan	2009	A3b_CC13
4	20	14	53	17	1	9	9	16	26	7	73	41	10	16	11	11	13	14	57	64	11	8	9	8	6	20	4	4	4	5	KZ_Sh_2021_42		1978	A3b_CC13
4	20	14	53	17	2	9	9	16	28	7	73	45	10	16	11	11	13	14	57	25	11	8	9	8	6	20	4	4	4	5	KZ_Sh_2021_151	Karaganda	2016	A3b_CC14
4	20	14	53	17	2	9	9	16	28	7	73	45	10	16	11	11	13	14	57	25	11	8	9	8	6	20	4	4	4	5	KZ_Sh_2021_152	Karaganda	2016	A3b_CC14
4	20	14	53	17	2	9	9	16	28	7	73	45	10	16	11	11	13	14	57	25	11	8	9	8	6	20	4	4	4	5	KZ_Sh_2021_153	Karaganda	2016	A3b_CC14
4	20	14	53	17	2	9	9	16	28	7	73	45	10	16	11	11	13	14	57	25	11	8	9	8	6	20	4	4	4	5	KZ_Sh_2021_154	Karaganda	2016	A3b_CC14
4	20	14	53	17	2	9	9	16	28	7	73	45	10	16	11	11	13	14	57	25	11	8	9	8	6	20	4	4	4	5	KZ_Sh_2021_155	Karaganda	2016	A3b_CC14
4	20	14	53	17	2	9	9	16	28	7	73	45	10	16	11	11	13	14	57	25	11	8	9	8	6	20	4	4	4	5	KZ_Sh_2021_156	Karaganda	2016	A3b_CC14
4	20	14	53	17	2	9	9	16	28	7	73	45	10	16	11	11	13	14	57	25	11	8	9	8	6	20	4	4	4	5	KZ_Sh_2021_157	Karaganda	2016	A3b_CC14
4	20	14	53	17	2	9	9	16	28	7	73	45	10	16	11	11	13	14	57	25	11	8	9	8	6	20	4	4	4	5	KZ_Sh_2021_158	Karaganda	2016	A3b_CC14
4	20	14	53	17	2	9	9	16	28	7	73	45	10	16	11	11	13	14	57	25	11	8	9	8	6	20	4	4	4	5	KZ_Sh_2021_159	Karaganda	2016	A3b_CC14
4	20	14	53	17	2	9	9	16	28	7	73	45	10	16	11	11	13	14	57	25	11	8	9	8	6	20	4	4	4	5	KZ_Sh_2021_160	Karaganda	2016	A3b_CC14
4	20	14	53	17	2	9	10	16	28	7	73	45	10	16	11	11	13	14	57	25	11	8	9	8	6	20	4	4	4	5	KZ_Sh_2021_150	Karaganda	2016	A3b_CC14
4	20	14	53	17	2	8	8	16	26	7	73	45	10	16	11	11	13	14	57	64	11	8	9	8	6	20	4	4	4	5	KZ_Sh_2021_66	East Kazakhstan	2004	A3b_CC15
4	20	14	53	17	2	8	8	16	26	7	73	45	10	16	11	11	13	14	57	64	11	8	9	8	6	20	4	4	4	5	KZ_Sh_2021_67	East Kazakhstan	2004	A3b_CC15
4	20	14	53	17	2	8	8	16	26	7	73	45	10	16	11	11	13	14	57	64	11	8	9	8	6	20	4	4	4	5	KZ_Sh_2021_68	East Kazakhstan	2004	A3b_CC15
4	20	14	53	17	2	8	8	16	26	7	73	45	10	16	11	11	13	14	57	64	11	8	9	8	6	20	4	4	4	5	KZ_Sh_2021_69	East Kazakhstan	2004	A3b_CC15
4	20	14	53	17	2	8	8	16	26	7	73	45	10	16	11	11	13	14	57	64	11	8	9	8	6	20	4	4	4	5	KZ_Sh_2021_72	East Kazakhstan	2004	A3b_CC15
4	20	14	53	17	2	8	8	16	26	7	73	45	10	16	11	11	13	14	57	64	11	8	9	8	6	20	4	4	4	5	KZ_Sh_2021_73	East Kazakhstan	2004	A3b_CC15
3	23	14	53	17	2	9	7	14	27	7	24	45	10	15	10	11	13	11	17	6		8	6	6	5	21	3	4	3	5	KZ_Sh_2021_173	Pavlodar	2016	B1_CC16
3	23	14	53	17	2	9	7	14	27	7	24	45	10	15	10	11	13	11	17	6		8	6	6	5	21	3	4	3	5	KZ_Sh_2021_174	Pavlodar	2016	B1_CC16
3	23	14	53	17	2	9	7	14	27	7	24	45	10	15	10	11	13	11	17	6		8	6	6	5	21	3	4	3	5	KZ_Sh_2021_175	Pavlodar	2016	B1_CC16
3	23	14	53	17	2	9	7	14	27	7	24	45	10	15	10	11	13	11	17	6		8	6	6	5	21	3	4	3	5	KZ_Sh_2021_176	Pavlodar	2016	B1_CC16
3	23	14	53	17	2	9	7	14	27	7	24	45	10	15	10	11	13	11	17	6		8	6	6	5	21	3	4	3	5	KZ_Sh_2021_177	Pavlodar	2016	B1_CC16
3	23	14	53	17	2	9	7	14	27	7	24	45	10	15	10	11	13	11	17	6		8	6	6	5	21	3	4	3	5	KZ_Sh_2021_178	Pavlodar	2016	B1_CC16
3	23	14	53	17	2	9	7	14	27	7	24	45	10	15	10	11	13	11	17	6		8	6	6	5	21	3	4	3	5	KZ_Sh_2021_179	Pavlodar	2016	B1_CC16
3	23	14	53	17	2	9	7	14	27	7	24	45	10	15	10	11	13	11	17	6		8	6	6	5	21	3	4	3	5	KZ_Sh_2021_180	Pavlodar	2016	B1_CC16
3	23	14	53	17	2	9	7	14	27	7	24	45	10	15	10	11	13	11	17	6		8	6	6	5	21	3	4	3	5	KZ_Sh_2021_181	Pavlodar	2016	B1_CC16
3	23	14	53	17	2	9	7	14	27	7	24	45	10	15	10	11	13	11	17	6		8	6	6	5	21	3	4	3	5	KZ_Sh_2021_182	Pavlodar	2016	B1_CC16
3	23	14	53	17	2	9	7	14	27	7	24	45	10	15	10	11	13	11	17	6		8	6	6	5	21	3	4	3	5	KZ_Sh_2021_183	Pavlodar	2016	B1_CC16
3	23	14	53	17	2	9	7	14	27	7	24	45	10	15	10	11	13	11	17	6		8	6	6	5	21	3	4	3	5	KZ_Sh_2021_184	Pavlodar	2016	B1_CC16
